# Structural Polymorphism of Chitin and Chitosan in Fungal Cell Walls From Solid-State NMR and Principal Component Analysis

**DOI:** 10.3389/fmolb.2021.727053

**Published:** 2021-08-25

**Authors:** Liyanage D. Fernando, Malitha C. Dickwella Widanage, Jackson Penfield, Andrew S. Lipton, Nancy Washton, Jean-Paul Latgé, Ping Wang, Liqun Zhang, Tuo Wang

**Affiliations:** ^1^ Department of Chemistry, Louisiana State University, Baton Rouge, LA, United States; ^2^ Department of Chemical Engineering, Tennessee Technological University, Cookeville, TN, United States; ^3^ Environmental Molecular Sciences Laboratory, Pacific Northwest National Laboratory, Richland, WA, United States; ^4^ Unité des Aspergillus, Département de Mycologie, Institut Pasteur, Paris, France; ^5^ Department of Microbiology, Immunology and Parasitology, Louisiana State University Health Sciences Center, New Orleans, LA, United States

**Keywords:** chitin, chitosan, solid-state NMR, fungi, cell wall, *Aspergillus*, *Candida*, principal component analysis

## Abstract

Chitin is a major carbohydrate component of the fungal cell wall and a promising target for novel antifungal agents. However, it is technically challenging to characterize the structure of this polymer in native cell walls. Here, we recorded and compared ^13^C chemical shifts of chitin using isotopically enriched cells of six *Aspergillus*, *Rhizopus*, and *Candida* strains, with data interpretation assisted by principal component analysis (PCA) and linear discriminant analysis (LDA) methods. The structure of chitin is found to be intrinsically heterogeneous, with peak multiplicity detected in each sample and distinct fingerprints observed across fungal species. Fungal chitin exhibits partial similarity to the model structures of α- and γ-allomorphs; therefore, chitin structure is not significantly affected by interactions with other cell wall components. Addition of antifungal drugs and salts did not significantly perturb the chemical shifts, revealing the structural resistance of chitin to external stress. In addition, the structure of the deacetylated form, chitosan, was found to resemble a relaxed two-fold helix conformation. This study provides high-resolution information on the structure of chitin and chitosan in their cellular contexts. The method is applicable to the analysis of other complex carbohydrates and polymer composites.

## Introduction

**FIGURE 1:**
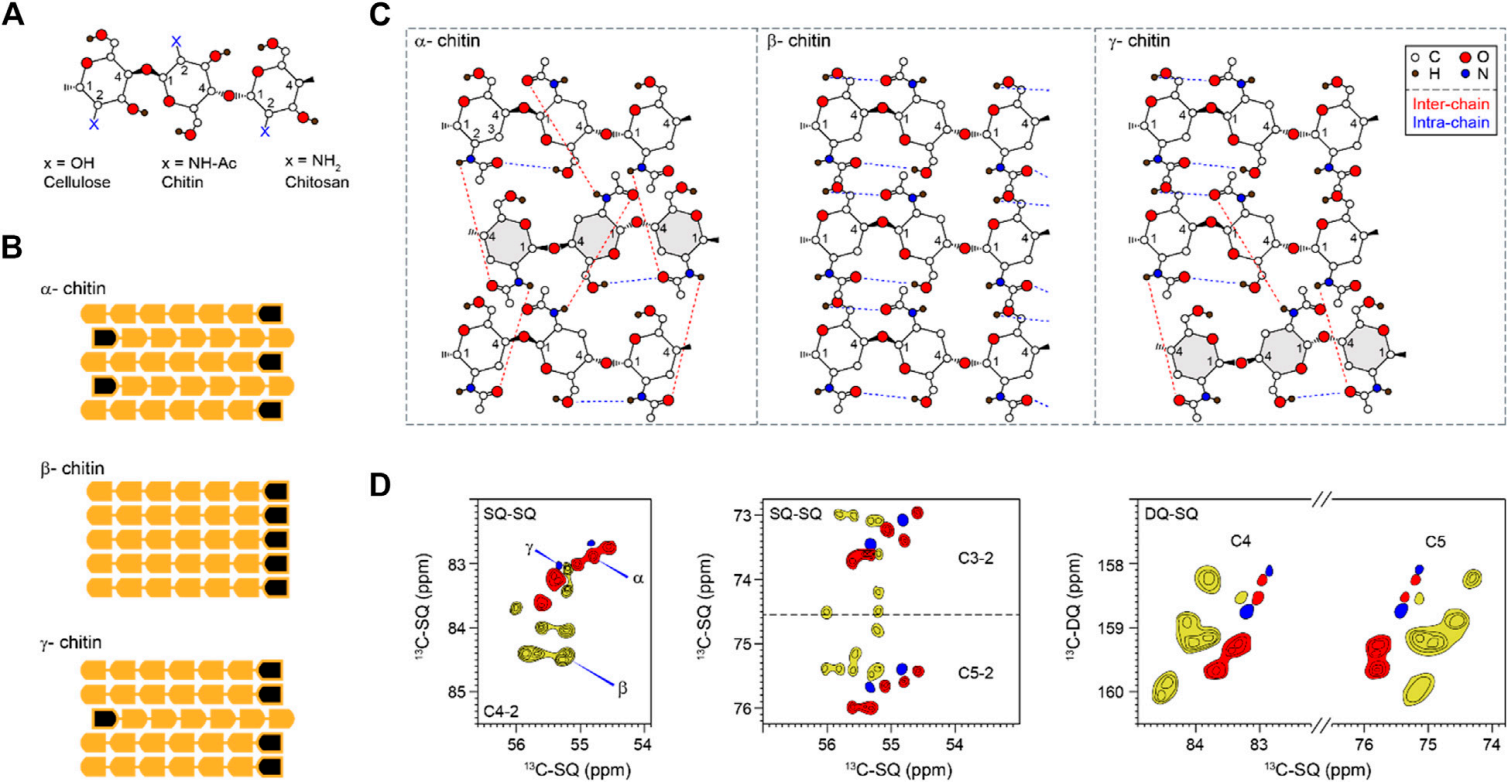
Representative structures and NMR signals of chitin. **(A)** Substitutions at the C2 position for chitin and chitosan. **(B)** Polymorphic types (α, β, and γ) of chitin showing different chain orientations. Black marks denote the non-reducing ends of chains. **(C)** Hydrogen-bonding patterns of different chitin allomorphs. Blue and red dash lines indicate intra-chain and inter-chain hydrogen bonds, respectively. The antiparallel chains in α and γ chitins are in grey. The hydroxyl at C3 is not shown to make the structure less complex. The structural schemes are adapted from (Rudall, 1963; Kameda et al., 2005; Sawada et al., 2012a). **(D)** 2D ^13^C-^13^C correlation spectra simulated using literature-reported chemical shifts on model samples (Supplementary Table S2). Representative C4-2 and C3/5-2 regions were shown for single-quantum (SQ)-SQ correlation spectra. The C4 and C5 region is also shown for a double-quantum (DQ)-SQ correlation spectrum. α, β, and γ are represented in red, yellow, and blue respectively. Contour lines represent the number of data sets used (and the number of overlapped peaks).

Chitin is the second-most abundant biopolymer in nature, only behind cellulose. Widely distributed in different organisms, chitin is often found as a supportive and protective component of the body armor (namely the exoskeleton) in arthropods and the cell walls of fungi and some algal species (Pillai et al., 2009; Rinaudo, 2006). The structures of chitin and its largely deacetylated form called chitosan have similarity to the organization of cellulose (Heux et al., 2000; Jarvis, 2003; Okuyama et al., 2000; Rinaudo, 2006; Saito et al., 1987). All these three polysaccharides are linear polymers of β-1,4-linked glucoses or their amide derivatives. Structurally, the hydroxyl group at position C-2 of a glucopyranose unit is replaced by an acetamido or an amino group, changing to the N-acetylglucosamine (GlcNAc) unit in chitin and the glucosamine (GlcN) residue in chitosan (Figure 1A). Chitin and chitosan, especially the latter, have also drawn tremendous attention due to their promising applications as polymer scaffolds for tissue engineering, wound dressing, drug delivery, and pharmaceuticals (Jayakumar et al., 2010).

The amide and carbonyl groups in chitins drive the formation of hydrogen bonds and crystalline fibrils. X-ray crystallography has reported three chitin allomorphs, with substantial variation in the chain orientation and the hydrogen-bonding pattern (Sikorski et al., 2009; Yui et al., 2007). Adjacent chains are packed in an antiparallel or parallel way in the α- and β-forms, respectively (Figure 1B). The third type of structure, γ-chitin, can be considered as a mixture of parallel and antiparallel packings, but sometimes it is treated simply as a variant of the α-allomorph (Rinaudo, 2006). The structure of α-chitin is stabilized simultaneously by intra-chain O-H…O and inter-chain N-H…O hydrogen bonding (Figure 1C) (Kameda et al., 2005; Deringer et al., 2016). The former is a hydrogen bond consistently observed in all three allomorphs. The latter is relatively rare in the γ-form and is absent in the β-chitin (Kameda et al., 2004; Sawada et al., 2012a; Sawada et al., 2012b). The coexistence of inter- and intra-chain hydrogen bonds has made α-chitin the most stable, ordered, and tightly packed structure, widely found in arthropods, Porifera, Bryozoa, and fungi (Ehrlich et al., 2007; Ehrlich et al., 2017). β- and γ-allomorphs are less common: the former can be found in diatoms and cephalopods, while the latter was reported in beetles and loligo species (Brunner et al., 2009; Kaya et al., 2017). The currently available information on chitin structure was obtained using highly crystalline materials isolated and purified mainly from marine sources. Although chitin is also a major fungal polysaccharide (Erwig and Gow, 2016; Gow et al., 2017), our understanding of its structural characteristics in the fungal cell wall remains inadequate.

Biochemical assays have revealed that chitin, β-glucan, and mannan are held together by covalent linkages in the human pathogen *Aspergillus fumigatus*, forming the core of the cell wall (Latge, 2007; Latgé and Chamilos, 2020). This structural module is resistant to alkali treatment and therefore has been proposed as the central scaffold of fungal cell walls (Latgé et al., 2017). Recently, we have employed high-resolution solid-state NMR methods to investigate the structure of biomolecules in the intact cells of *A. fumigatus* (Kang et al., 2018; Zhao et al., 2020). Unexpectedly, we identified three major types (and in total eleven subtypes) of GlcNAc units, as resolved from their distinct ^13^C and ^15^N chemical shifts, which are indicators of structural variations (Kang et al., 2018). These chitin forms were found to be extensively associated with each other inside chitin microfibrils as shown by their strong inter-residue interactions. These findings have unveiled the surprisingly high structural polymorphism of chitin in its cellular environment and raised three unresolved questions related to the chitin structure. First, is the structure of chitin in the fungal cell wall similar to the crystallographic structures determined using standard samples? Second, is there any dependence between the chitin structure and the fungal type? Third, is chitin structure modulated by external stresses such as antifungal drugs and hypersaline environments?

To answer these questions, we compared the ^13^C chemical shifts of chitins identified in the cells prepared from three *Aspergillus* species (*Aspergillus fumigatus, A. nidulans,* and *A. sydowii*)*, Rhizopus delemar*, and two *Candida* pathogens (*C. albicans* and *C. auris*), following exposure to various antifungal drugs and salt concentrations. All these fungal species investigated here are significant human pathogens causing life-threatening infections in immunodeficient individuals and known to display different chitin composition in their cell walls (Brown et al., 2012; Latge and Calderone, 2006). Root mean square deviation (RMSD) heatmap, principal component analysis (PCA), and linear discriminant analysis (LDA) of chemical shifts were performed for the comparison of 62 chitin forms. Most fungal chitins align well with literature-reported α- and γ-allomorphs but deviate substantially from the β-form. The structure of chitin proved robust, remaining unaffected even under high salinity or in the presence of antifungal drugs, caspofungin and amphotericin B (AmB). In addition, chitosan was also identified in *R. delemar* and *A. sydowii*. Comparison of the literature-reported and our observed chemical shifts showed that most chitosan molecules are closely related to the Type-II salt model compound that has a relaxed two-fold conformational structure. This study presents a widely applicable research strategy for evaluating the structure of cellular carbohydrates and provides the structural basis for developing chitin-targeting antifungal agents.

## Materials and Methods

### Preparation of ^13^C, ^15^N-Labeled Fungal Cells

In total, nine ^13^C,^15^N-labeled samples were prepared for six fungal species including *A. fumigatus, A. nidulans, A. sydowii, C. albicans*, *C. auris,* and *R. delemar* following a recently established protocol (Kirui et al., 2019)*.* To examine the potential effect of antifungal drugs on chitin structure, three parallel batches were prepared for *A. fumigatus:* without drug, with caspofungin (2.5 µg/ml: above the minimum inhibitory concentration), and with AmB (2.5 µg/ml). To examine if salt concentration and osmotic pressure affect chitin structure, two batches of materials were prepared for the seawater inhabitant *A. sydowii*, with 0.5 and 2.0 M NaCl to represent optimal and high salinity conditions, respectively (Perez-LIano et al., 2020). Briefly, uniformly ^13^C,^15^N-labeled materials were obtained by culturing the fungi in modified minimum liquid media containing ^13^C-glucose as the sole carbon source. The nitrogen sources are different for various fungal species, with ^15^N-sodium nitrate for *A. fumigatus* and *A. nidulans*, ^15^N-ammonium nitrate for *A. sydowii*, and ^15^N-ammonium sulfate for *C. albicans, C. auris,* and *R. delemar*. All these species are able to grow on inorganic nitrogen sources and were cultivated alternatively on ammonium or nitrate salts. The cultures were incubated at the optimum temperatures of 25–31°C for respective fungal species. The culture duration was 3 days for *A. fumigatus, A. nidulans*, *R. delemar*, *C. albicans*, and *C. auris*, and 7 days for *A. sydowii*. Fungal materials were then collected by centrifugation at 7,000 × g for 20 min. The harvested fungal pellets were washed thoroughly using phosphate buffered saline (pH 7.4) to remove small molecules and reduce the ion concentration. For each sample, approximately 30 mg of the hydrated whole-cell material was packed into a 3.2 mm magic-angle spinning (MAS) rotor for solid-state NMR characterization.

### Solid-State NMR Experiments

All the high-resolution solid-state NMR data were collected on a Bruker 800 MHz (18.8 Tesla) Bruker Avance III HD spectrometer at the National High Magnetic Field Laboratory (Tallahassee, FL) and a Varian VNMRS 850 MHz (19.9 Tesla) spectrometer at the Environmental Molecular Sciences Laboratory (EMSL; Richland, WA). The experiments were conducted in 3.2 mm MAS HCN probes under 12–13.5 kHz MAS at 290–293 K. The ^13^C chemical shifts were externally referenced to the adamantane CH_2_ signal at 38.48 ppm on the tetramethylsilane scale. The ^15^N chemical shifts were referred externally through the methionine nitrogen peak (127.88 ppm) in the model peptide formyl-Met-Leu-Phe (MLF). Typical ^1^H radiofrequency field strengths 50–83 kHz and 50–62.5 kHz for ^13^C. The ^13^C chemical shifts were recorded using the 2D Dipolar-Assisted Rotational Resonance (DARR) experiment with a 100-ms mixing time and the 2D ^13^C-^13^C COmbined $\text{R}2_{n}^{v}$ -Driven (CORD) sequence with a 53-ms mixing time (Hou et al., 2013). 2D ^15^N-^13^C N(CA)CX heteronuclear correlation spectra were measured to detect chitin amide signals (Pauli et al., 2001). The N(CA)CX spectrum was recorded using a 0.6-ms ^1^H-^15^N cross polarization (CP), a 5-ms ^15^N-^13^C CP contact, and a 100-ms DARR mixing time. The experimental and processing parameters used for 2D ^13^C-^13^C and ^13^C-^15^N spectra are summarized in Supplementary Table S1. Resonance assignment was facilitated by comparison with previously reported chemical shifts indexed in a carbohydrate database (Kang et al., 2020), which distinguish chitin from glucans and other nitrogenated polysaccharides. To compare the chemical shift differences in different chitin forms observed in fungi and from different model samples, a heat map was constructed from the root-mean-square deviation (RMSD) values calculated using the comparison of the literature-reported and observed chemical shifts with normalization by the total number of carbon atoms in a monomer (i.e., 8 for chitin carbons of C1-C6, CO, and CH_3_). Similar approaches are also used for comparing different forms of fungal chitin. Good correlations give low RMSD values.

### Principal Component Analysis and Linear Discriminant Analysis

We conducted PCA to facilitate the analysis of the species- and condition-dependent data of chitin chemical shifts. PCA is a form of multivariate analysis employed to reduce the many correlated variables to just a few new variables (the principal components) that describe most of the variation in a dataset. Recently, PCA has been successfully employed to provide valuable insights on chemical shift data for small molecules (Tasic et al., 2002) and proteins (Kazumasa and Goto, 2007; Sakurai et al., 2019). The PCA was first conducted using MATLAB for the entire dataset from both the available literature and freshly measured spectra (Supplementary Tables S2, S3). A 62 × 8 matrix was composed, with each row representing a different chitin form identified in the NMR spectra, and each column corresponding to the chemical shifts observed for a ^13^C atom at a particular location in the chitin structure. Similarly, PCA was also run separately for three subsets of chitin chemical shift data to compare 1) only the data from fungal chitin, 2) drug-free and drug-treated samples, and 3) optimal and high salinity conditions. For each PCA, a singular value decomposition (SVD) analysis was performed on the data matrix to generate orthogonal eigenvectors with values known as “loadings” or “PCA coefficients” arranged in a matrix by column. Loadings are normalized and used to describe the contribution made by each chemical shift, while the magnitude of the eigenvector shows how much of the variance in the data is explained by each eigenvector. The largest eigenvector defines the axis principal component 1 (PC1), and the next largest one defines PC2, etc. Each NMR dataset can be given a score based on the loadings and is projected onto the principal axes to show how the chemical conditions in that sample affect the observed chemical shifts. Samples of molecules within similar chemical environments are expected to cluster together in the “PC-space” if the dimension-reduction is successful. Because loadings describe a linear combination of the original variables, the relationship between the mean-centered data, score, and loadings are the matrix product: [PC score] = [data] × [PC loadings].

In addition, we performed linear discriminant analysis (LDA) to identify the factor that distinguishes the chitins produced in *Candida* species and other fungi. LDA was performed on the PCA scores, which provide linear discriminant (LD) loadings and LD scores. The scores of observations in separate classes fall approximately into a normal distribution with as little overlap with other classes as possible. The addition of more classes requires additional linear discriminants. Similar to PCA, the relationship between LD scores and LD loadings is: [LD score] = [data] × [LD loadings].

## Results and Discussion

### Solid-State NMR Fingerprints of Chitin in Fungal Cell Walls

Solid-state NMR has been widely applied to differentiate the hydrogen-bonding patterns, identify the type of chitin, and determine the degree of acetylation of chitin and chitosan (by tracking the intensities of CO and CH_3_ peaks) in model samples (Tanner et al., 1990; Heux et al., 2000; Kameda et al., 2004; Kono, 2004; Kasaai, 2010; King et al., 2017). The spectroscopic signatures of model chitin allomorphs are summarized in 2D^13^C-^13^C correlation spectra simulated and plotted using literature-reported chemical shifts (Supplementary Table S2) (Jang et al., 2004; Kono, 2004; Tanner et al., 1990; Brunner et al., 2009; Kaya et al., 2017; Kolbe et al., 2021) (Figure 1D). α-chitin has its C3 and C5 peaks distributed as two separated regions (72–73.7 and 75.4–76 ppm like a doublet) while most β-chitins have characteristic C3 and C5 signals sharply clustered in the 74–76 ppm region. The signals of γ-chitin are mixed with those of α- and β-allomorphs, with better alignment to the α-form. The same trend is retained in the double-quantum (DQ)-SQ correlation spectrum. The INADEQUATE spectrum, with an example shown in Supplementary Figure S1, was not explicitly used in this study but have been frequently measured for characterizing cellular samples.

**FIGURE 2:**
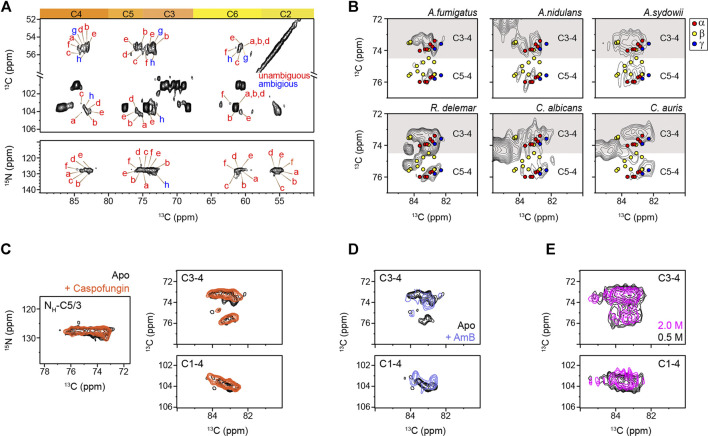
Peak multiplicity of chitin in different fungi. **(A)** Representative signals of different chitin types in *A. fumigatus*. ^13^C-^13^C (top) and ^15^N-^13^C (bottom) correlation spectra resolved different forms of chitin molecules. Chitin forms with all carbon sites unambiguously resolved are labeled in red (types a–f), while the ambiguous forms are in blue (types g and h), with the ambiguous (partially resolved) carbon sites underlined. **(B)** Comparison of chitin signals in different fungi. The C5-C4 and C3-C4 regions are shown. Colored dots denote the data from three crystalline forms of chitin: α-chitin (red), β-chitin (yellow), and γ-chitin (blue). **(C)** 2D ^15^N-^13^C and ^13^C-^13^C correlation spectra of *A. fumigatus* without drug (apo; black) and with caspofungin treatment (orange). **(D)** 2D ^13^C-^13^C spectra of *A. fumigatus* without drug (apo; black) and with amphotericin B (AmB; blue). (**E**) Overlay of 2D ^13^C-^13^C correlation spectra collected on two *A. sydowii* samples cultured with 0.5 M NaCl (black) and 2 M NaCl (magenta).

Different from the model compounds, analysis of cellular systems using solid-state NMR spectroscopy has remained challenging due to the coexistence of a large variety of biomolecules, whose signals often exhibit significant overlap (Poulhazan et al., 2018; Narasimhan et al., 2019; Kelly et al., 2020; Zhao et al., 2020; Reif et al., 2021). Fortunately, the presence of nitrogen in the amide group has made chitin chemically unique among the structural polysaccharides in the cell wall. At the same time, the nitrogenated sugars in the intracellular content have already been filtered out using CP-based methods, which remove the signals of mobile sugars but selectively highlight the stiff molecules in the cell wall. The ^15^N chemical shifts (∼128 ppm) and the unique ^13^C chemical shift of the nitrogen-linked carbon 2 (54–56 ppm) are the characteristic signals of chitin for initiating the resonance assignment. High-resolution 2D ^13^C-^13^C and ^15^N-^13^C correlation spectra collected on freshly prepared *A. fumigatus* mycelia resolved the signals of six major types of chitins (type a–f), together with two forms with some carbon sites being ambiguously assigned (types g and h) (Figure 2A; Supplementary Figure S2). The ^13^C full width at half maximum (FWHM) linewidth is in the range of 0.5–0.7 ppm for the chitin in native cell walls.

The C5-C4 and C3-C4 cross-peaks showed comparable spectral patterns among the three *Aspergillus* samples, indicative of structural similarity (Figure 2B). *R. delemar*, however, had more extensive signals in this spectral region due to its uniquely high content of chitin and chitosan molecules (Melida et al., 2015; Ghormade et al., 2017; Lecointe et al., 2019). The spectra of *C. albicans* and *C. auris* looked alike, but their spectral patterns differ from the other filamentous fungi studied. Comparing to α and γ chitin, the characteristic signals of β-chitin were less overlapped with the spectra of all the fungal samples. Chains in β-chitin are arranged in a parallel way, with only intramolecular H-bonds. This results in a unique and less tightly packed structure for β-chitin, which is swollen in water and exhibiting high reactivity. Most of the literature-reported chemical shifts (Supplementary Table S2) from the α-allomorph are enclosed in the spectral envelope of the fungal samples studied here. Still, the expected signals of β-chitin mostly fell out of the spectral region.

Caspofungin inhibits the β-1,3-glucan synthesis, but when above the minimal inhibitory concentration, it causes a paradoxical effect enhancing the production of chitin to compensate for the loss of β-1,3-glucan (Loiko and Wagener, 2017). Consistently, the intensities of chitin peaks were enhanced relative to other cell wall components (Supplementary Figure S3), but no major changes were observed in the chemical shifts (Figure 2C). Therefore, the increased amount of chitin has insignificant effects on the structure of this molecule. Similarly, the addition of AmB that targets ergosterol in fungal membranes (Anderson et al., 2014) only redistributed the intensities among chitin subtypes without inducing new signals (Figure 2D) The robustness of the chitin structure is further confirmed by the comparable signals observed in the saprophytic *A. sydowii* samples cultured with either optimal or high salinities (Figure 2E) (Perez-LIano et al., 2020). Although chitin structure altered moderately among different fungi, it remained resistant to these external stresses (Supplementary Tables S4, S5). These observations are not surprising because AmB and caspofungin do not directly target chitin. Nikkomycin is the most notable chitin synthesis inhibitor and is thus of significant interest for further investigations (Steinbach and Stevens, 2003; Nix et al., 2009; Li et al., 2019). Recently combinatorial biosynthetic approaches have been used integrating echinocandin and chitin inhibitors which show potent antifungal activity (Li et al., 2019).

### Comparison of Chitin Structures Using Chemical Shift Analysis

**FIGURE 3:**
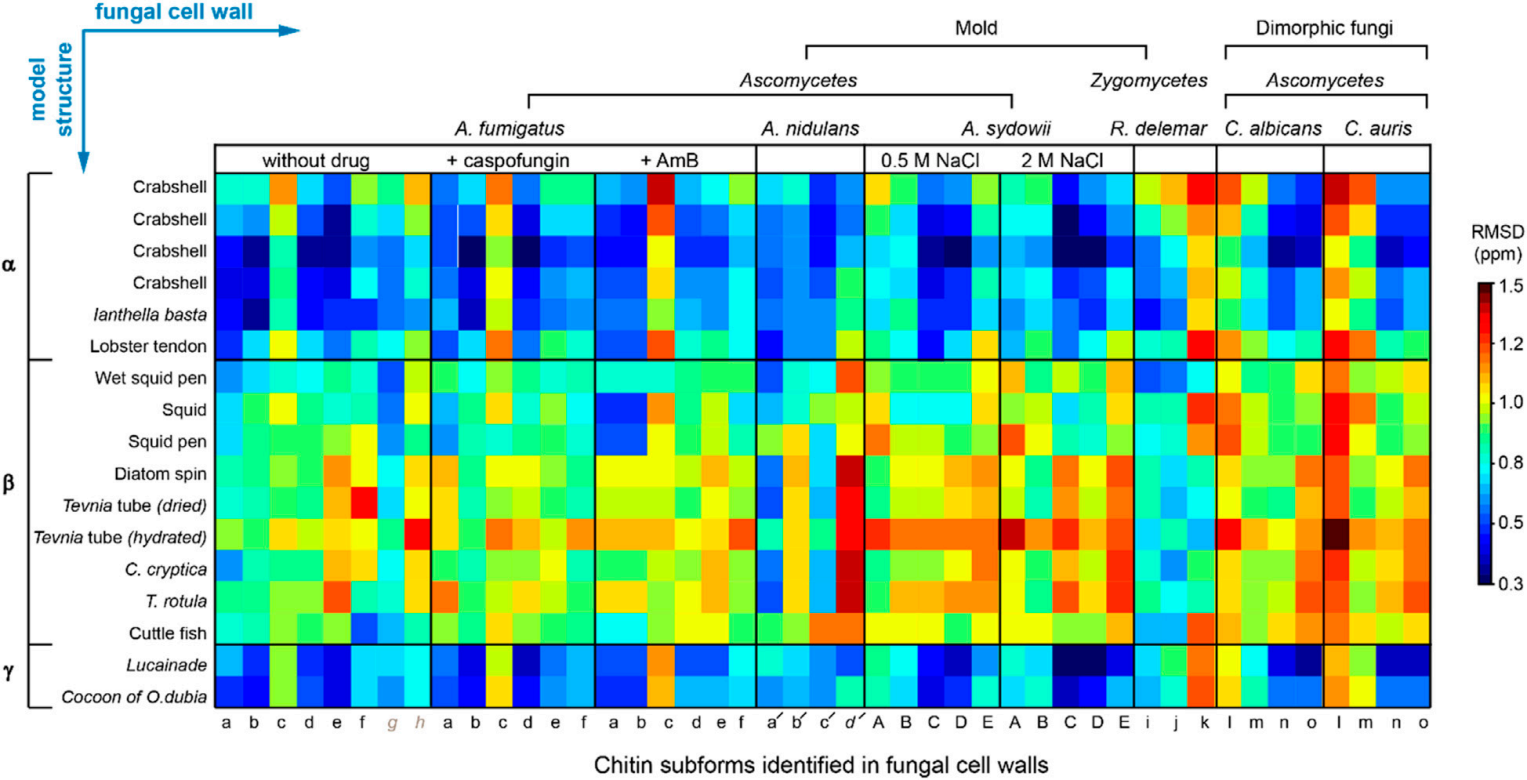
^13^C chemical shift RMSD map comparing chitin structure. Data were compared between the observed 45 chitin forms in nine fungal cell walls (*x*-axis) and the three crystalline forms reported by literature (*y*-axis). Data from six fungal species were shown, including three species of *Ascomycetes* (*A. fumigatus*, *A nidulans* and *A. sydowii*), a sample from Zygomycetes (*R. delemar*), and two Ascomycetes yeast species (*C. albicans* and *C. auris*). Most chitin types showed similarity to α-chitin form. The color scale is shown, with units of ppm. Good correlation with RMSD less than 0.5 ppm (within NMR linewidth) are in dark blue. The forms with certain ambiguous carbon sites are labeled in italics and grey. The chemical shift values used for the analysis are provided in Supplementary Tables S2, S3.

We compared the ^13^C chemical shifts obtained on the 45 chitin forms in nine fungal samples (Supplementary Table S3) with the 17 datasets reported in the literature (Supplementary Table S2) (Jang et al., 2004; Kono, 2004; Tanner et al., 1990; Brunner et al., 2009; Kaya et al., 2017; Kolbe et al., 2021), generating a chemical shift RMSD heatmap (Figure 3). The 45 subforms identified and assigned in the intact fungal cell wall include eight chitin forms (a–h) in drug-free *A. fumigatus*, six forms (a–f) in each of the two *A. fumigatus* samples treated with either caspofungin or amphotericin B, four chitin forms (a′-d′) in *A. nidulans*, five forms (A–E) in each of the two *A. sydowii* samples cultured with 0.5 M or 2 M NaCl, three chitin forms (i,k) in *R. delemar*, and four chitin forms (l-o) in each of the two *Candida* samples. Each of the 765 comparisons was represented by an RMSD value based on 16 ^13^C chemical shifts of C1-C6, CO, and CH_3_ from two different chitin forms. Similar methods have been used to compare the NMR data collected on other fibrillar biomolecules including cellulose and amyloid fibrils (Elkins et al., 2016; Wang et al., 2016; Qiang et al., 2017). We found that fungal chitin correlated relatively well with α-chitin. Small RMSD values below the spectroscopic resolution (0.5 ppm) were observed for some datasets of *A. fumigatus* and *C. albicans*. Reasonable correlations between the cell wall chitin and the γ-chitin model structure were also noted, which can be understood by treating γ-chitin as a derivative of α-chitin due to their structural similarities. In contrast, β-chitins failed to correlate with our observations, with large RMSD typically in the range of 0.7–1.6 ppm. Exceptions were observed for *R. delemar* (Figure 3), suggesting the formation of structurally unique chitin domains in this fungus.

**FIGURE 4:**
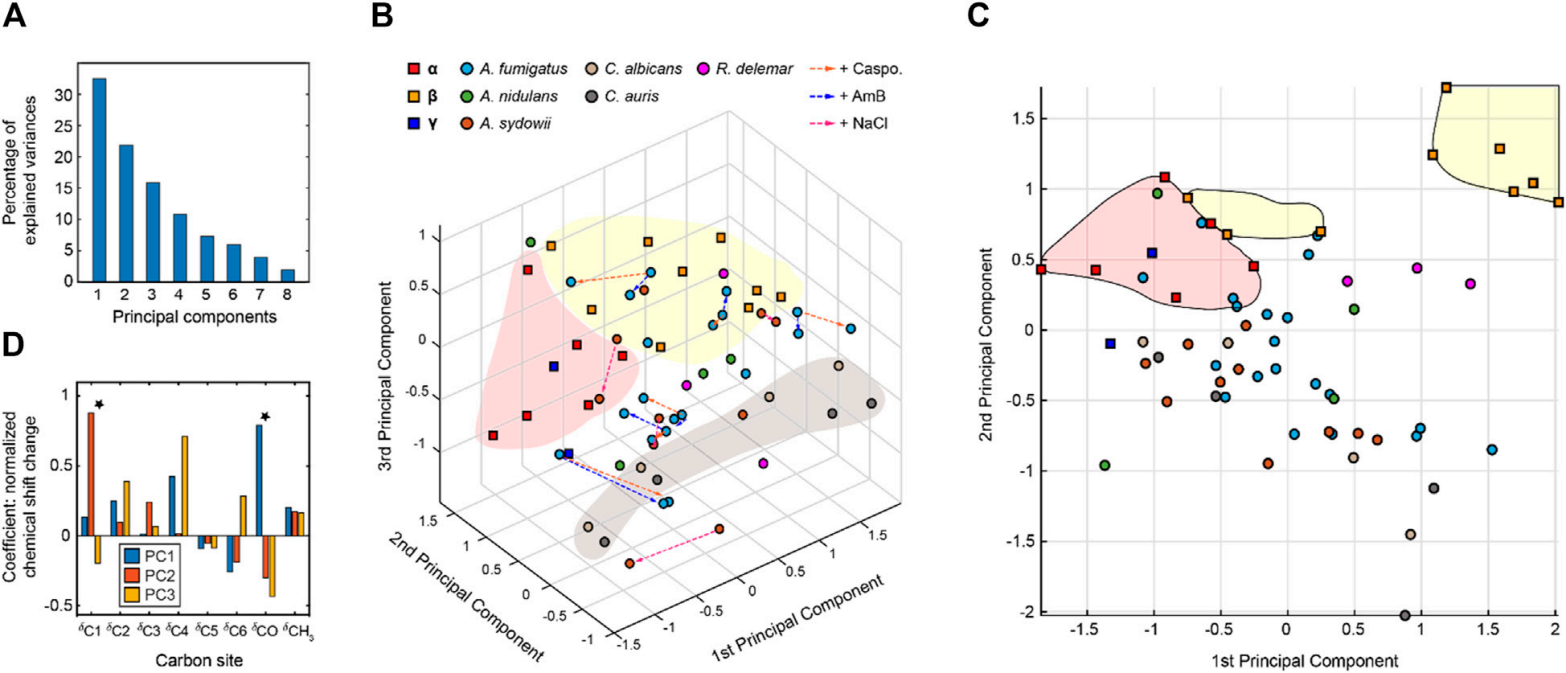
Principal component analysis of chitin chemical shifts. **(A)** Variance explained by each principal component (PC). **(B)** PCA scores for chitin NMR chemical shifts projected onto principal component 1 (PC1) vs. PC2 vs. PC3. Model chitin allomorphs (α, β, and γ-types) are shown using squares while chitin forms identified in fungal cell walls are presented using circles. Shaded regions in red and yellow are used to enclose α- and β-type chitins, respectively. The shaded region in grey mainly contains data from *Candida* species. Data from different model samples and fungal species are color-coded. Arrows in orange, blue, and magenta represents the changes induced by caspofungin (Caspo.), the amphotericin B (AmB), and NaCl (from 0.5 to 2.0 M), respectively. **(C)** PCA scores of chitin chemical shifts projected onto PC1 and PC2 proving a better visualization of most chitin forms. **(D)** Loadings for each PC. Asterisks indicate the most pronounced differences for PC1 and PC2.

The NMR chemical shift data were subjected to PCA. As a dimension-reduction analysis tool, a useful PCA result necessitates that the importance of each consecutive PC declines rapidly. PCs are constructed by the SVD algorithm in an unsupervised manner, beginning with a new axis that maximizes the variance of all data points when projected onto it, then constructing orthogonal axes according to the same criteria. The eigenvectors returned from the SVD calculation are shown in Figure 4A, with the sum normalized to 100, showing the percent of variance in the data explained by each PC. With the first three PCs explaining 70% of the variance in the data, a safe majority of the variance is now explained in those three variables, and the first three PCs should be able to account for the major factors contributed to the chemical shift.

The 3D PCA score plot composed using the first three PCs (Figure 4B) illustrates the relationship between each chitin sample in the PC space. Consistent with the heatmap representation, principal component 1 (PC1) primarily differentiated the α and β chitin standards, with the γ-chitin standards more closely associated with the former. This is more clearly recognizable in the 2D presentation of PC1 vs. PC2 (Figure 4C), that the spreading of α and β chitins are on the negative side and positive sides of PC1, respectively. We only observed a relatively small amount of stretching of β-chitins to the negative side. In addition, γ-chitin are distributed mostly to the α-chitin side. Therefore, it is likely that PC1 can sense the difference in hydrogen bonding and chain-packing. This is confirmed by the loadings where the first principal component experiences the most significant change at the carbonyl group (Figure 4D). Together, PC1 and PC2 can clearly resolve most forms of β-chitins as a self-isolated group. *Candida* chitins and β-chitins show up on the two extreme positions of PC2, with scores distributed somewhat evenly between −1 and 1 of PC2 and PC3.

The PCA loadings shown in Figure 4D are the weight given to each original variable (chemical shifts) in the linear combination that defines each PC, from which one can gather the relative magnitude and direction (as indicated by the sign) of change in those variables expected to occur over positive displacement in the respective PC score. The loadings show that while PC1 is mostly concerned with the carbonyl, PC2 focuses on the C1 atom, while PC3 and PC1 focus on C4 atom that also (together with C1) participates in the glycosidic linkages of chitin molecule.

To only focus on fungal chitin, we conducted a separate PCA by excluding the data from α, β, and γ model allomorphs (Supplementary Figure S4). PCA scores for all fungi chitins indicate that similarities between chitins within a single fungal species are sparse, as many allomorphs of the same species can be found at opposite extremes of both PC1 and PC2, accounting together for almost 60% of variation. Two other PCAs were conducted to respectively focus on the effect of drug and salt conditions (Supplementary Figures S5, S6). It should be noted that the changes caused by antifungal drugs and increased salinity are trivial when compared with the large structural dispersion of native chitin molecules.

**FIGURE 5:**
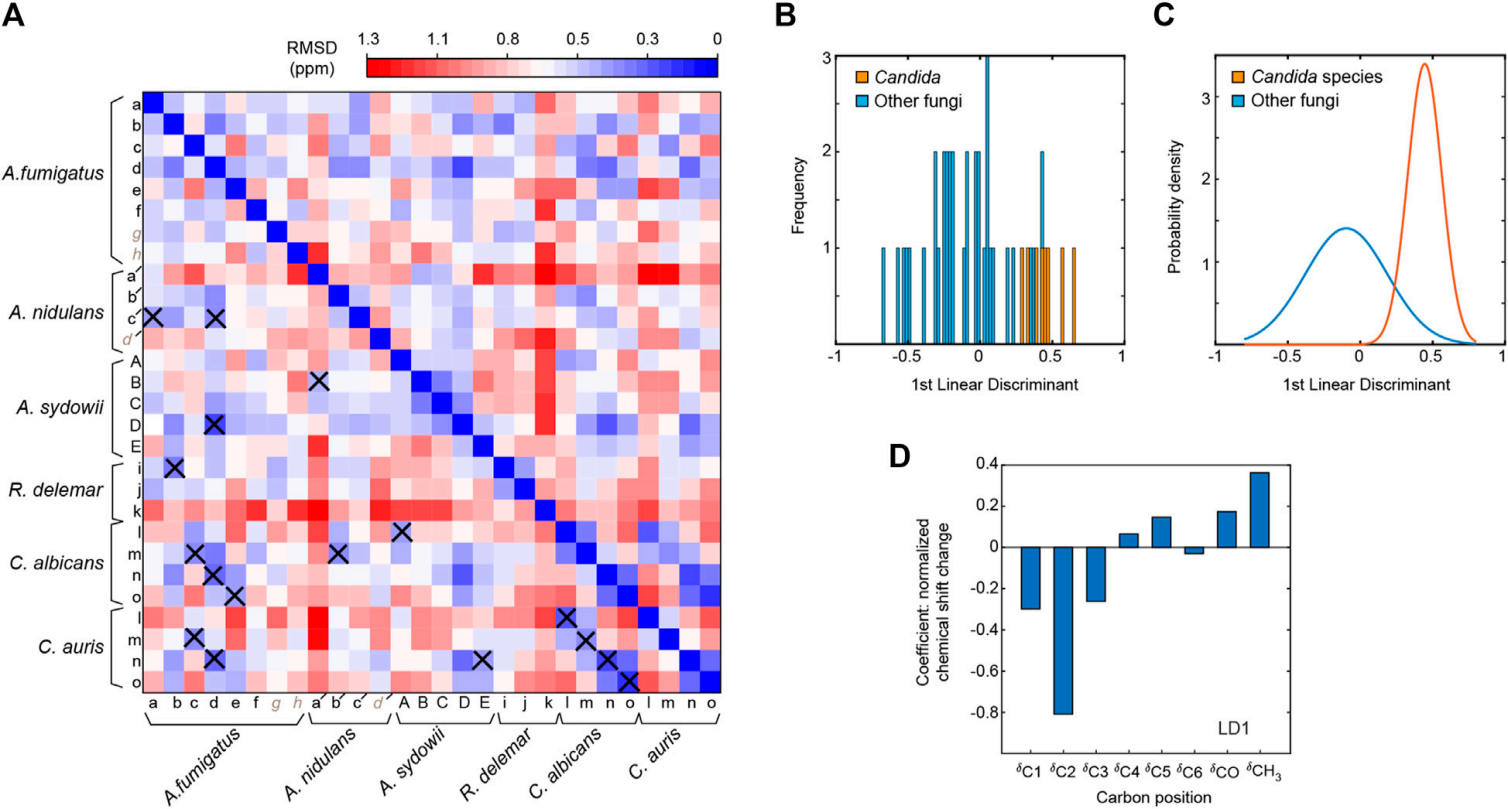
Comparison of chitin forms identified in different fungal species. **(A)** Chemical shift RMSD heatmap comparing the chitin forms observed in different fungi. Good correlations with RMSD of less than 0.5 ppm are highlighted using crosses. **(B)** Linear discriminant analysis with *Candida* fungi (*C. albicans* and *C. auris*) classified differently from other fungal species, with linear discriminant 1 (LD1) scores shown in a histogram. This panel mainly shows the frequency in which LD1 scores fall into a particular range (the width of each bar). **(C)** Gaussian probability distributions of LD1 scores. The *Candida* data falls into a smaller range than the other fungi, therefore, there is a much higher probability that a *Candida* species will fall near their statistical mean. **(D)** LD1 loadings corresponding to the chemical shifts of each carbon site.

In addition, partial structural similarities were noted for some chitin subtypes residing in different fungal strains (Figure 5A). For *A. fumigatus*, a few reasonably good correlations can be found with *A. nidulans* and *A. sydowii, Candida* species, and *R. delemar*. These observations revealed the partial alignment of chitin structure in different species. The best correlation was found between the type-d chitin of *A. fumigatus* and the type-D form of *A. sydowii*, with a small RMSD (0.19 ppm) well below the NMR linewidth. Just like the *Aspergillus* samples, *R. delemar* is also a filamentous fungus, but it exhibited only a single modest correlation with *Aspergillus* species, indicating the structural uniqueness of the chitin produced in *R. delemar.*

The *Candida* samples prepared in this study were grown only as a yeast form. The two *Candida* species are highly similar to each other, with small RMSD values (0.16–0.32 ppm) when comparing each type of chitin between two *Candida* species. For example, the RMSD is 0.16 for the comparison of type-m chitins in *C. albicans* and *C. auris*. The RMSD is similarly good for the comparisons of type-n (0.21 ppm) and type-l (0.26 ppm) chitins, and only slightly larger for the type-m form (0.32 ppm). In contrast, the filamentous fungi (*Aspergillus* and *Rhizopus* species) studied here only exhibited partial similarities to the *Candida* species. It is possible that filamentous fungi require for their hypha a specific form of chitin because the strength to hold the tube-shaped mycelium should be different and stronger than holding a balloon shape like a yeast.

The results also aligned with the number and families of chitin synthase (CHS) genes seen in these species. In yeasts (*Candida* and *Saccharomyces* for example), 3 to 4 CHS genes have been encountered belonging to the families I, II and IV. In *Aspergillus* and *Rhizopus*, however, 9 to 23 genes have been found and they not only belong to the three classes (I, II and IV) that were also identified in yeasts, but also have contributions from additional classes (III, V, VII, VI or VIII) (Lenardon et al., 2007; Ma et al., 2009; Muszkieta et al., 2014).

To directly identify the structural factor that differentiates the chitin types in yeasts and filamentous fungi, we conducted linear discriminant analysis (LDA). Different from the PCA method described above, LDA is a supervised learning method. LDA can pinpoint the variables that distinguish between the observations that have already been arranged into classes by their properties of interest. Here, we categorized the data into two separate classes to distinguish *Candida* strains (grown as yeasts) from other fungal species (grown as mycelium), which produced a linear discriminant (Figure 5B). Their probability distributions (Figure 5C) only overlapped slightly, and the loadings (Figure 5D) indicated that *Candida* chitin and the chitins of other fungal species could be best distinguished by the chemical shifts of C2 and CH_3_, thus revealing the key sites for tracking fungal chitin structure.

The results provided three structural implications. First, the structure of chitin is highly polymorphic in fungal cell walls. At this moment, it is unclear whether the observed polymorphism is related to the diverse groups of chitin synthases involved in the biosynthesis of this polymer, which should be further investigated using functional genomics and spectroscopic approaches. It also raised a major question on the individual function of all the CHS genes (>20 genes in the *Zygomycetes*). This study raises unanswered questions about the function of the different classes of chitin synthases in the cell wall structuration. Based on the ssNMR data presented here it does suggest that all CHS synthesized a chitin with very similar structure. The actual biological role of each CHS should be totally dependent on the cellular localization of each synthase in the cell wall as recently suggested (Walker et al., 2013).

Second, the model structures of α-chitins, as characterized using the highly crystalline material isolated and purified from marine sources, are remarkably preserved among different fungi. This is intriguing as the interactions with other polysaccharides, often by covalent linkages in fungal cell walls (Gow et al., 2017), did not substantially perturb the structure of chitin. This result agrees with the low number of linkages identified biochemically in the β-1,3-glucan-chitin core of *A. fumigatus* cell wall and the poor growth phenotype resulting from the deletion of the CRH genes coding for the glycosyltransferases that are responsible for forming glucan-chitin linkages (Latgé et al., 2017). It is a supplementary argument to suggest that these chitin-glucan covalent connections might not be structurally important for the building of the cell wall.

Third, the structure of chitin is resistant to environmental stimuli, such as non-chitin-focused drug treatment as well as hypersaline environment and osmotic pressure. The structural robustness of chitin and its central role in mechanically supporting the cell wall confirmed the suitability of chitin as a potential target for the development of novel antifungal compounds. It also indicated that the increase in chitin concentration in the cell wall is a survival response, which is not depending on the stress proposed. At this moment, it remains unknown how to reconcile the microscopic structure of the different chitin microfibrils seen in electron microscopy (Lenardon et al., 2007; Lenardon et al., 2010; Muszkieta et al., 2014) with the atomic level ssNMR data.

### Spectroscopic and Structural Features of Fungal Chitosan

**FIGURE 6:**
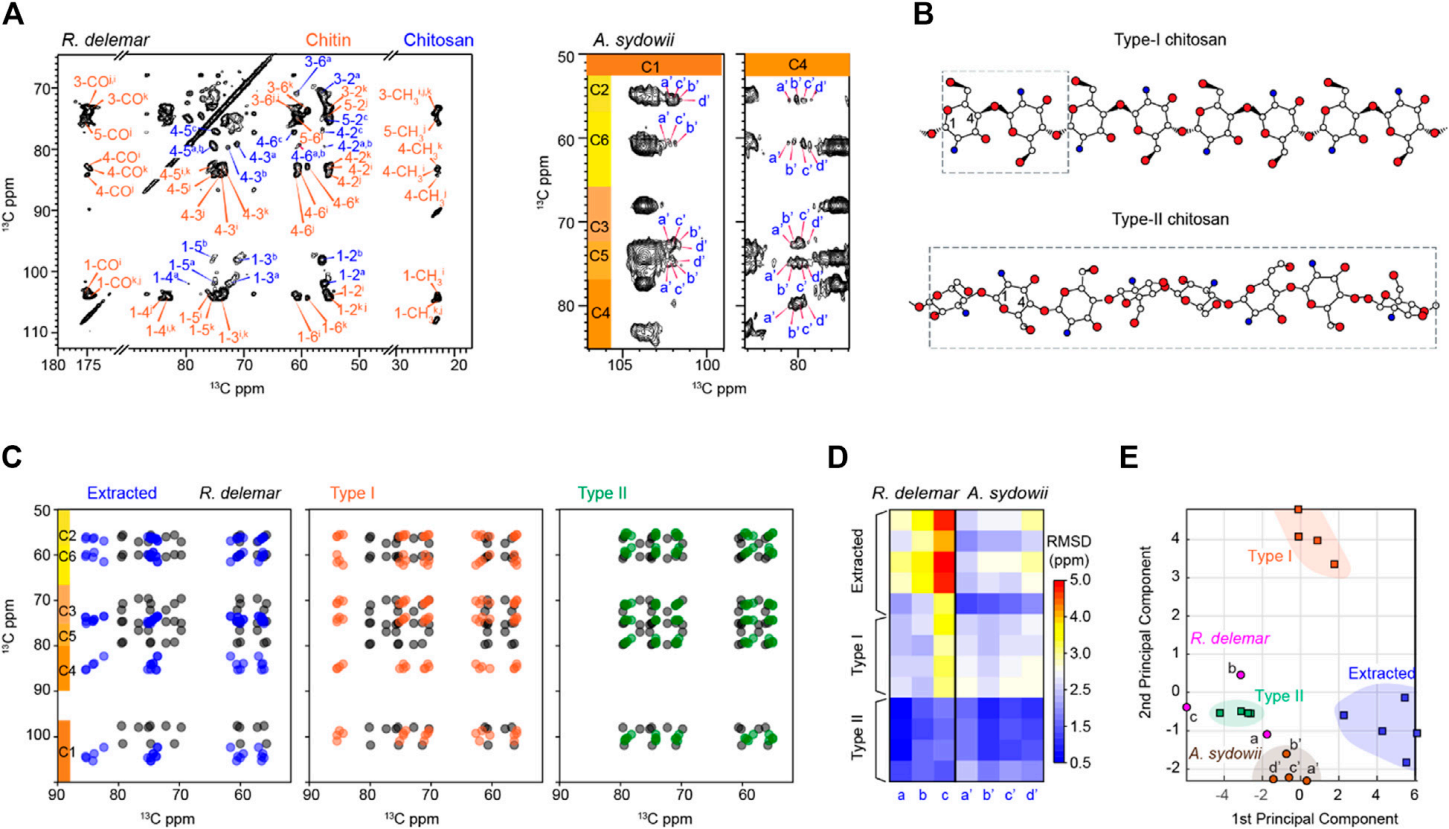
*R. delemar* and *A. sydowii* cell walls are rich in chitosan. **(A)** Representative 2D ^13^C-^13^C CORD spectrum of *R. delemar* and *A. sydowii* cells showing many sets of chitosan signals (blue). **(B)** Representative structures of Type-I and Type-II chitosan molecules. Nitrogen (blue), oxygen (red), and carbon (white) atoms are shown but hydrogen atoms are not included for simplicity. The repeating units are shown in dash line boxes. Structure schemes are adapted from (Okuyama et al., 2000). **(C)** Simulated spectra of *R. delemar* chitosan (black) overlaid with the literature-reported chitosan forms including extracted chitosan **(blue; left panel)**, Type-I salts with inorganic acids **(orange; middle panel)**, and Type-II salts with inorganic acids** (green; right panel)**. **(D)** ^13^C chemical shift RMSD map for the comparison between fungal cell wall chitosan (*X*-axis) and model samples (*Y*-axis). The color scale unit is ppm. **(E)** PCA scores of chitosan. The data analyzed include Type-I (orange squares) and Type-II (green squares) salts with inorganic acids, extracted chitosan (blue square), as well as the chitosan forms identified in *R. delemar* (magenta circles) and *A. sydowii* (brown circles).

Deacetylation of chitin leads to chitosan. Chitosan exists in a semicrystalline form in solids but can be solubilized by acidic solutions. In the fungal cell wall, chitosan has been proposed to serve as a backbone to bind other biomolecules, such as dityrosines or melanin (Chrissian et al., 2020a; Chrissian et al., 2020b). The NMR signals of chitosan are resolved from those of chitin by the absence of CH_3_ and CO peaks at 22 and 174 ppm (Supplementary Figure S7). The substantial modification in the chemical structure and the hydrogen-bonding patterns induce unique chemical shifts at most carbon sites as shown by Figure 6A. The structures of two major chitosan forms, Types I and II salts with inorganic acids, have been reported (Figure 6B), which exhibited different helical conformations (Saito et al., 1987; Ogawa et al., 2004; Franca et al., 2008). Type-I chitosan has a fully extended two-fold helical structure. The repeating unit of type-II chitosan is four times longer than that of type-I, with a relaxed two-fold helix and a tetrasaccharide repeat in a helical asymmetric unit. Overlay of the spectra predicted using the chemical shifts available in the literature and our dataset revealed that *R. delemar* chitosan could not structurally align with those extracted from various sources such as crab tendon, crab shell, and shrimp shell (Figure 6C). The same discrepancy was also present for the Type-I compound, but a better correlation was observed with the Type-II structure.

No chitosan signal was observed in these fresh *A. fumigatus* samples. This is in agreement with a recent genomic study which indicates that the deletion of all deacetylase genes in *A. fumigatus* does not lead to any significant growth phenotype (Mouyna et al., 2020). Interestingly, the occurrence of a significant amount of chitosan in xerophilic *Aspergillus* species may indicate that the fungus synthesizes chitosan to make the cell wall more flexible to fight against the increase in osmotic pressure.

The type-c chitosan in *R. delemar* exhibited bad correlations with the chitosan prepared using extracted chitin (RMSD ∼5 ppm) and Type-I chitosan in inorganic salt. RMSD values as large as that should be originated from totally different structures. In contrast, the type-c chitosan correlated reasonably with Type-II chitosan (RMSD <1.5 ppm) (Figure 6D). Similar trends were observed for the other two types (a and b) of chitosan molecules. For example, comparison of chitosan-a in *R. delemar with* Type-II model structures gave very small RMSDs of 0.6–0.8 ppm. The results indicate that chitin chitosan differs from the extracted forms or the Type I structure, but closely resembles the Type-II structure. This trend was projected in the RMSD heatmap of ^13^C chemical shifts for both *R. delemar* and *A. sydowii* (Figure 6D). In the PCA plot, chitosan signals were separated remarkably well by the first two principal components, which account for 89% of the variation in the data (Figure 6E; Supplementary Figure S8). *R. delemar* and *A. sydowii* samples shared more in common with the Type-II chitosan standards but lacked structural similarity to the Type-I standard and extracted chitosan. Therefore, chitosan in the fungal cell wall only has moderate correlations to the Type-II standard structure.

It should be noted that the RMSD values between different chitosan forms are substantially larger than those calculated for chitin. The NMR data actually suggest a new type of chitosan structure that is different from those previously characterized. It is also intriguing that chitosan molecules in extracted materials and intact fungal cell walls are structurally distinct. A possible reason is the solubilization and extraction procedures used in previous studies might have restructured this molecule before subjection to structural characterization. For example, alkali treatment was known to induce chitin deacetylation. The distinct organization of molecules in arthropods and fungi, as well as the potential difference in the degree of deacetylation (Liu et al., 2019), might also contribute to the observed discrepancy. This differs from the case of chitin, which is an insoluble polymer and often found in the crosslinked core of fungal cell walls, thus being more resistant to isolation and processing procedures. More in-depth investigations are needed to identify the biochemical reason driving the structural complexity of chitosan and to fully understand its function-related structures in fungal cell walls.

## Conclusion

The high-resolution dataset enabled by solid-state NMR spectroscopy has made it possible to analyze and compare the structural features of cell wall polysaccharides using statistical approaches. Such protocols will accommodate the rapidly expanding ssNMR dataset and open new research avenues related to the structural investigations of cellular and extracellular biomolecules as well as natural and artificial biomaterials (Arnold et al., 2018; Bougault et al., 2019; Kang et al., 2019; Ehren et al., 2020; Kelly et al., 2020). The polymorphic structure of chitin and its resistance to external stress was determined in fungal species of biomedical and environmental significance. This information has the potential to facilitate the development of antifungal strategies targeting the unique structures of chitin or its biosynthesis.

## Data Availability

The datasets presented in the study are included in the article and Supplementary Material. Additional data can be requested from the corresponding author.
