# Supplementary material for: Structural Polymorphism of Chitin and Chitosan in Fungal Cell Walls From Solid-State NMR and Principal Component Analysis
Source: Front Mol Biosci. 2021 Aug 25;8:727053. doi: 10.3389/fmolb.2021.727053 (PMC8423923; doi:10.3389/fmolb.2021.727053)
Supplement: Supplementary file 1 [file DataSheet1.pdf]

# Supplementary Material

## Structural Polymorphism of Chitin and Chitosan in Fungal Cell Walls from Solid-State NMR and Principal Component Analysis

Liyanage D. Fernando[1,†], Malitha C. Dickwella Widanage[1,†], Jackson Penfield[2], Andrew S. Lipton[3], Nancy Washton[3], Jean-Paul Latgé[4,&], Ping Wang[5], Liqun Zhang[2], Tuo Wang[1,*]

[1] Department of Chemistry, Louisiana State University, Baton Rouge, LA, United States

[2] Department of Chemical Engineering, Tennessee Technological University, Cookeville, TN, United States

[3]Environmental Molecular Sciences Laboratory, Pacific Northwest National Laboratory, Richland, WA, United States

[4]Unité des Aspergillus, Département de Mycologie, Institut Pasteur, Paris, France

[5] Departments of Microbiology, Immunology and Parasitology, Louisiana State University Health Sciences Center, New Orleans, LA, United States

[&] Current address: Institute of Molecular biology and Biotechnology, University of Crete, Heraklion, Greece

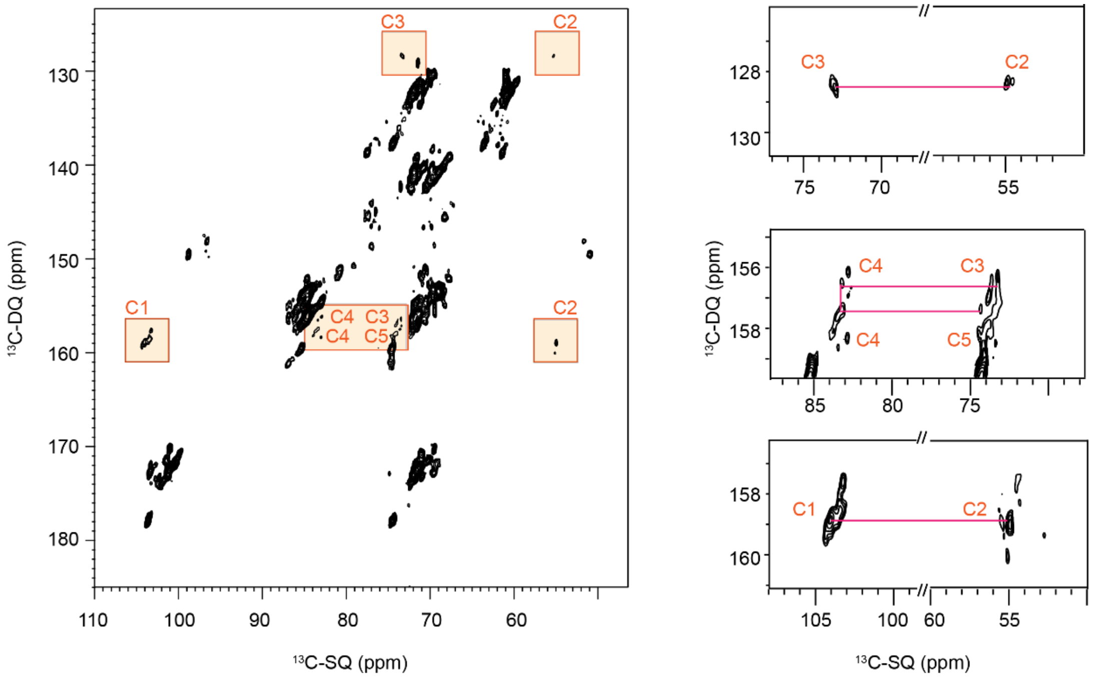


**Supplementary Figure S1**. 2D $^{13}$C-$^{13}$C DQ-SQ spectra of *A. fumigatus*. The full spectra (left) and selected regions of chitin signals (right) are shown. Chitin carbon peaks are labeled, and the carbon connectivity are shown in magenta lines.

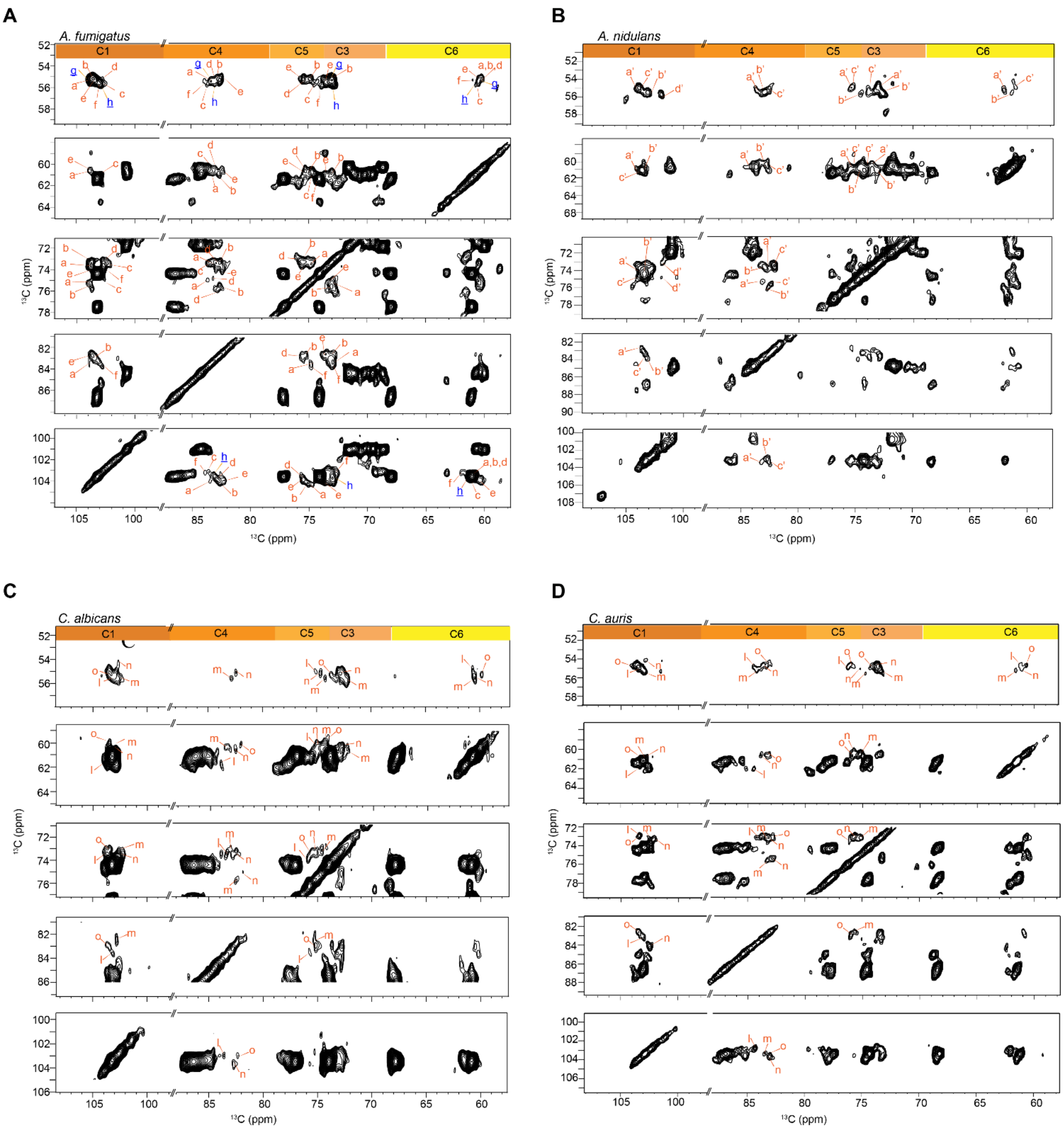


**Supplementary Figure S2.** 2D $^{13}C$-$^{13}C$ correlation spectra resolving chitin polymorphs in different fungi including **(A)** *A. fumigatus*, **(B)** *A. nidulans*, **(C)** *C. albicans*, and **(D)** *C. auris*. $^{13}C$-$^{13}C$ correlation spectra detect intramolecular interaction of chitin peaks. Different chitin forms are annotated using alphabetic letters and ambiguous chitin types are labeled in blue and ambiguous carbon sites are underlined.

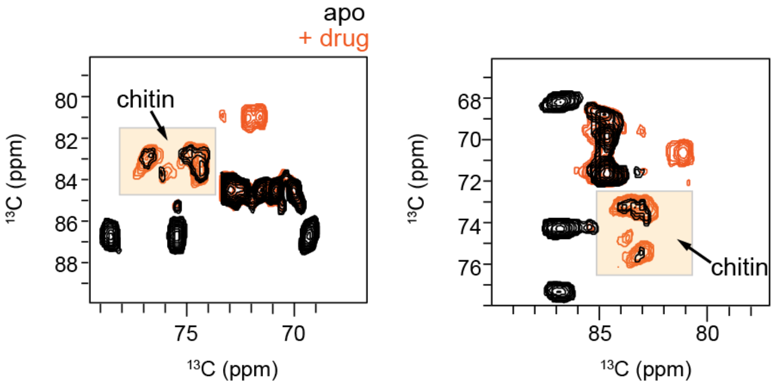


**Supplementary Figure S3.** 2D $^{13}$C-$^{13}$C correlation spectra of *A. fumigatus* showing the increased content of chitin after treatment by caspofungin. The chitin signals are highlighted using boxes in light orange, which have higher intensity in the drug-treated sample.

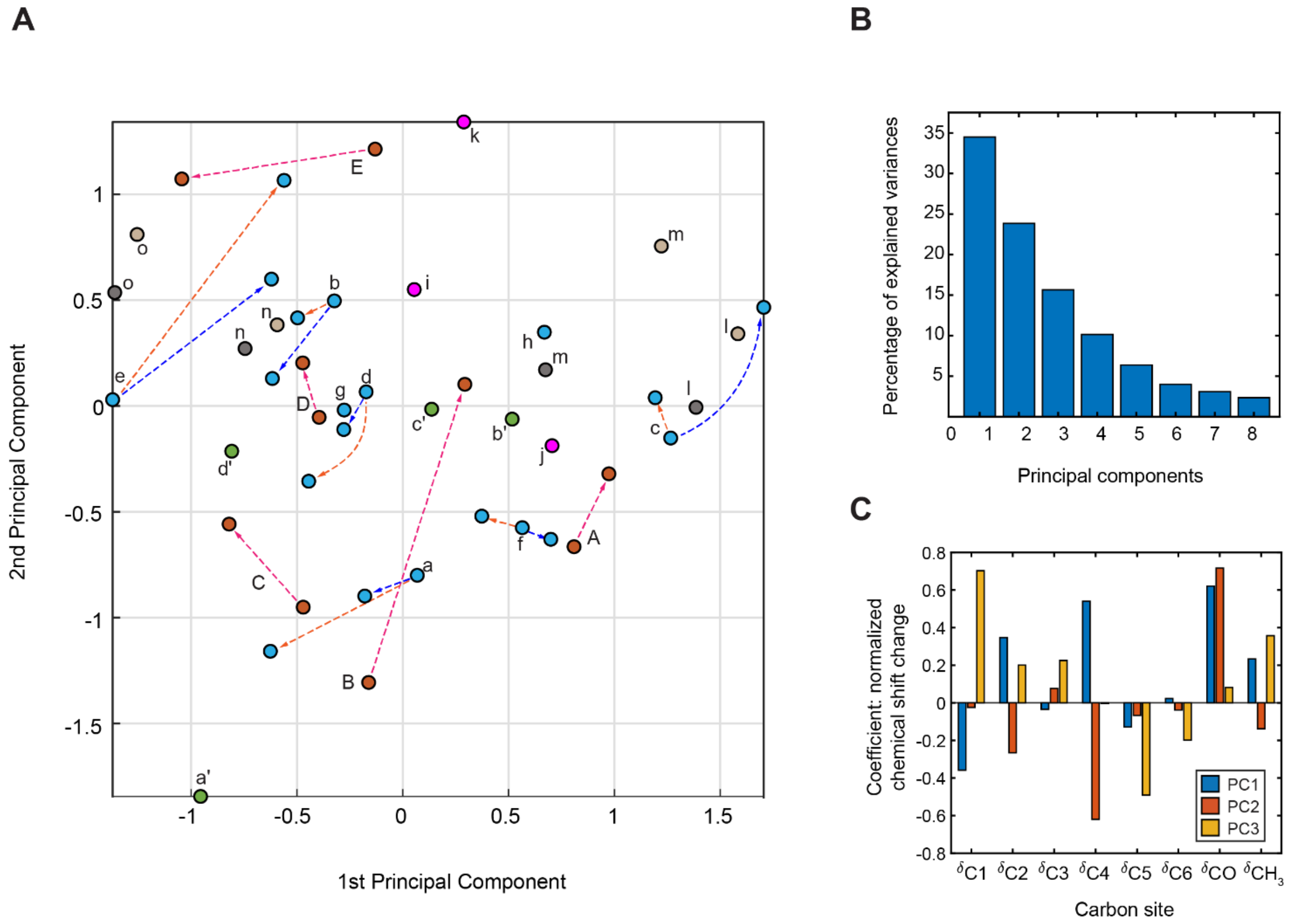


**Supplementary Figure S4.** PCA of different fungal chitin forms. (**A**) PCA scores of fungal species and their respective allomorphs. The color code is kept the same as **Fig. 4**. *A. sydowii* allomorphs are shown at different salt concentrations, and *A. fumigatus* allomorphs are shown in the presence and absence of antifungal drugs. (**B**) Variance explained by each principal component. (**C**) Loadings for the PCA results. Both PC1 and PC2 are defined by a CO shift in the same direction, while the C4 shift contributes in the opposite direction. PC3 and PC1 likewise share an inverse relationship to C1.  The results delineated how the chemical shift difference in different chitin forms.

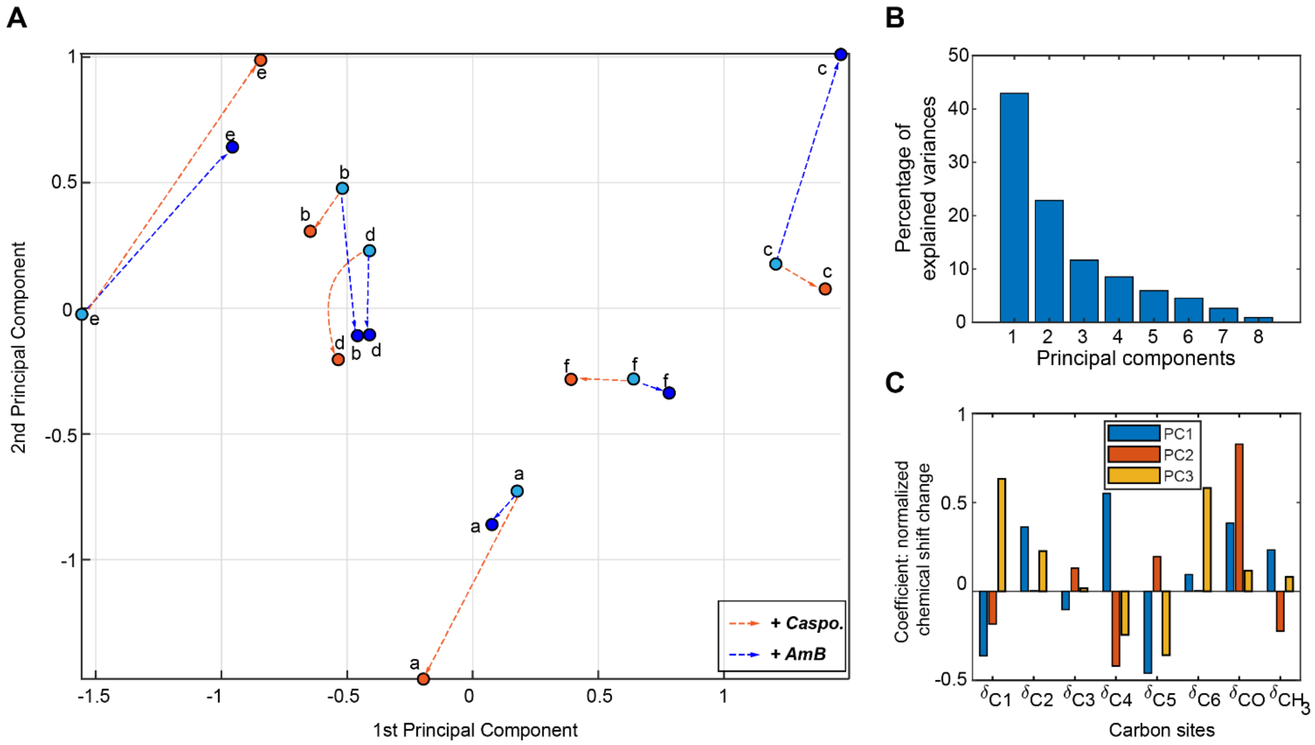


**Supplementary Figure S5.** PCA scores of *A. fumigatus* chitin with and without antifungal drugs. (**A**) PCA scores of the control sample (light blue), caspofungin-treated culture (orange), and amphotericin B-containing sample (dark blue) are shown. Arrows in orange and blue represent the changes induced by the caspofungin and AmB, respectively. The antifungal drug effect appears inconsistent and subtle from the perspective of the two dominant PC's. (**B**) Variance explained by each principal component. PC1 and PC2 account for almost 66% of the variation. (**C**) PCA loadings.

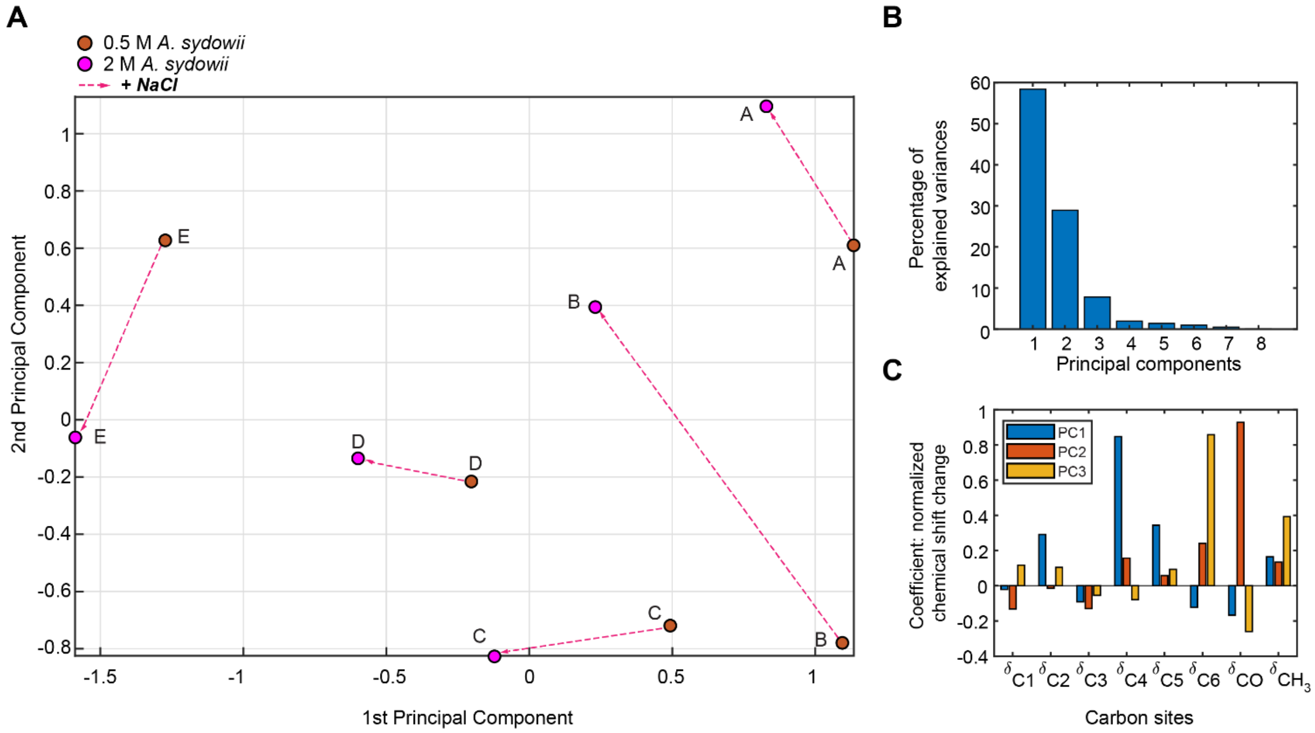


**Supplementary Figure S6.** Effects from hypersaline conditions. (**A**) PCA scores of *A. sydowii* chitin allomorphs at different salt concentrations. Each allomorph is coded labeled. Arrows in magenta represent the changes induced by a higher concentration of NaCl (from 0.5 M to 2.0 M). PC1 and PC2 scores are dominated by the differences between chitin allomorphs from E to D, to C, to B, then to A with PC1 increasing, rather than the salt concentration. Scores are distributed at the extreme ranges in both PC1 and PC2 for different allomorphs, while salt concentration caused only limited changes. (**B**) Variance explained by each principal component. The SVD of the matrix produced 8 PCs with the first 2 PCs being able to describe 87% of the variance. (**C**) PCA loadings. The effect of increasing salt concentration has a small but consistent effect on the chitin structure with respect to PC1: increasing the salt concentration decreases each chitin's PC1 score, which is mostly defined by the $\delta_{C4}$ chemical shift. Increasing the salt concentration has varied effects on the PC2 score, which is mostly defined by the carbonyl shift as indicated by PC2 loadings.

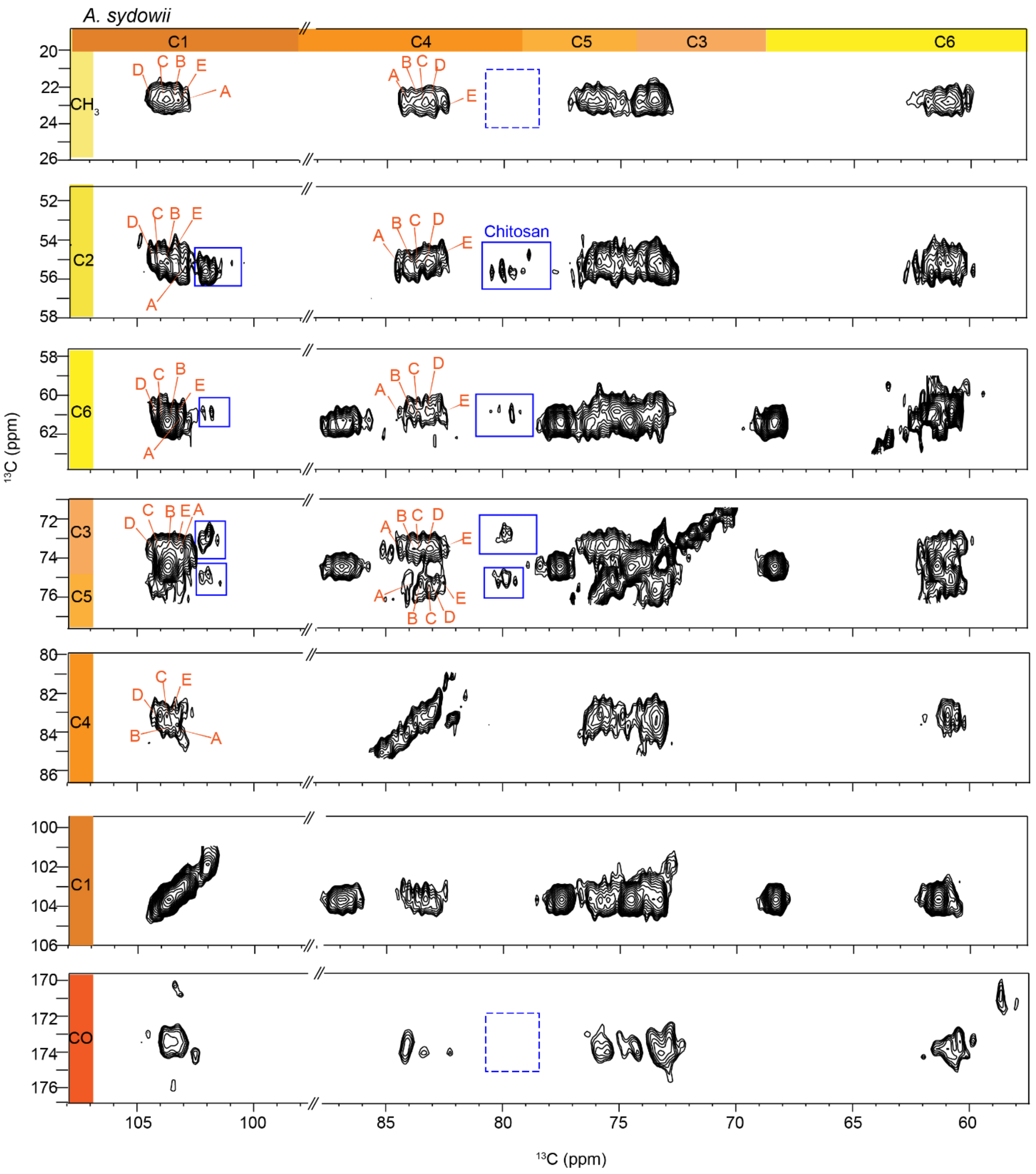


**Supplementary Figure S7.** 2D $^{13}C$-$^{13}C$ correlation spectra resolving chitosan signals in *A. sydowii*. These $^{13}C$-$^{13}C$ correlation spectra detect intramolecular cross-peaks. All chitin types are labeled with letters A-E. Blue boxes indicate the chitosan peaks, absence of $CH_3$ and CO peaks at 22 and 174 ppm (F1 dimension) confirms the chemical structure of these molecules.

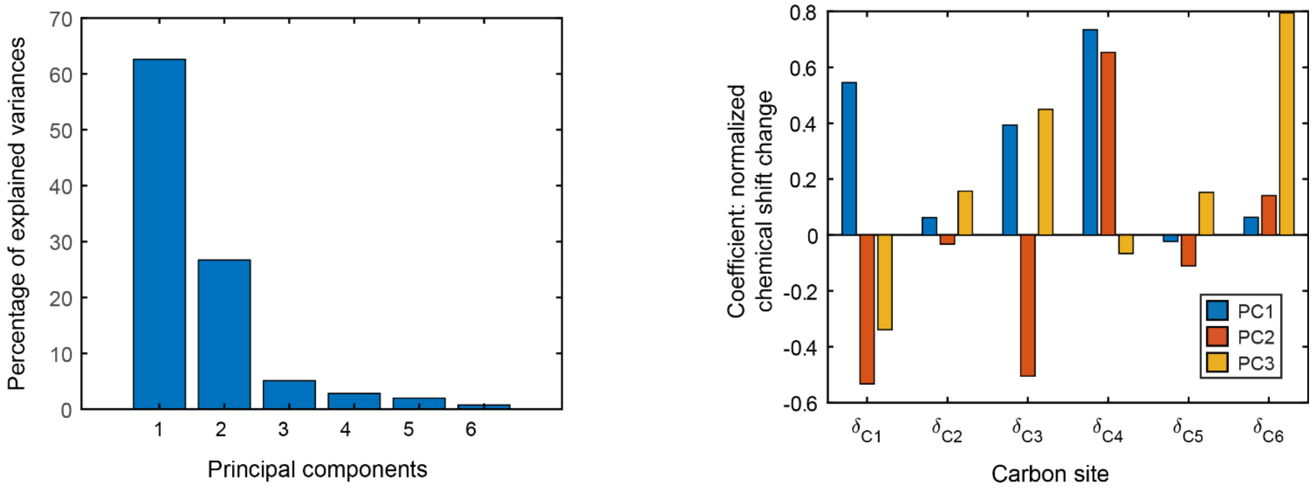


**Supplementary Figure S8.** Information related to the PCA scores of *A. sydowii* chitosan allomorphs. Variance explained by each principal component (left), and the PCA loadings (right) are shown. The first 2 PCs describe 87% of the variance.

**Supplementary Table S1:** Experimental and processing parameters of 2D ssNMR. 2D CP $^{13}C$-$^{13}C$ and $^{13}C$-$^{15}N$ correlation experiments allowed to resolve rigid chitin intramolecular peaks. The experimental parameters include the $^{1}H$ Larmor frequency, total experiment time (t), recycle delay (d1), number of scans (NS), The number of points for the direct (td2) and indirect (td1) dimensions, the acquisition time of the direct dimension (aq2) and the evolution time of indirect dimension (aq1), spectral width (sw1 and sw2), mixing time ($t_m$), increment delay (IN_F). The processing parameters include the window function and associated parameters.

| | | | Acquisition parameters | | | | | | | | | | | Processing parameters | |
|---|---|---|---|---|---|---|---|---|---|---|---|---|---|---|---|
| Sample | Experiment | $\omega_{0, 1H}$ (MHz) | t (h) | d1 (s) | NS | td2 | td1 | aq2 (ms) | aq1 (ms) | sw2 (ppm) | sw1 (ppm) | $t_m$ (ms) | IN_F (µs) | Window function | Parameters |
| ***A. fumagitus*** | | | | | | | | | | | | | | | |
| *W/o drug* | CORD | 800 | 4.2 | 1.7 | 16 | 2400 | 560 | 17.9 | 7.2 | 332.8 | 191.1 | 53 | 26 | QSINE | SSB 6 |
| | N(CA)CX | 800 | 1.3 | 1.7 | 16 | 2400 | 180 | 17.9 | 9.0 | 332.8 | 123.3 | 30 | 100 | GM | LB -5.0, GB 0.1 |
| +*Caspo* | CORD | 800 | 4.2 | 1.7 | 16 | 2400 | 560 | 17.9 | 7.2 | 332.8 | 191.1 | 53 | 26 | QSINE | SSB 6 |
| | N(CA)CX | 800 | 1.3 | 1.7 | 16 | 2400 | 180 | 17.9 | 9.0 | 332.8 | 123.3 | 30 | 100 | GM | LB -5.0, GB 0.1 |
| +AmB | CORD | 850 | 9.1 | 2 | 32 | 2496 | 512 | 24.9 | 5.1 | 233.9 | 167.1 | 53 | 20 | QSINE | SSB 4, Tdeff 1400(F2) |
| ***A. nidulans*** | | | | | | | | | | | | | | | |
| | DARR | 850 | 9.1 | 2 | 32 | 2496 | 512 | 24.96 | 5.1 | 233.9 | 167.1 | 100 | 20 | QSINE | SSB 2.8, Tdeff 1400(F2) |
| ***Candida* sp.** | | | | | | | | | | | | | | | |
| *C.albicans* | CORD | 800 | 3.2 | 1.5 | 16 | 2400 | 490 | 17.9 | 7.1 | 332.8 | 171.3 | 53 | 29 | QSINE | SSB 2.6 |
| *C.auris* | CORD | 800 | 3.2 | 1.5 | 16 | 2400 | 490 | 17.9 | 7.1 | 332.8 | 171.3 | 53 | 29 | QSINE | SSB 4 |
| ***A. sydowii*** | | | | | | | | | | | | | | | |
| 0.5 M NaCl | DARR | 850 | 4.5 | 2 | 16 | 2496 | 512 | 24.96 | 5.1 | 233.9 | 167.1 | 100 | 20 | QSINE | SSB 3 |
| 2 M NaCl | DARR | 850 | 4.5 | 2 | 16 | 2496 | 512 | 24.96 | 5.1 | 233.9 | 167.1 | 100 | 20 | QSINE | SSB 3 |

**Supplementary Table S2:** $^{13}C$ chemical shifts (ppm) of chitin microfibrils (α-chitin, β-chitin, γ-chitin) and chitosan molecules from literature. Not applicable (/).

| Type | C1 | C2 | C3 | C4 | C5 | C6 | CO | CH3 | Sources | References |
|---|---|---|---|---|---|---|---|---|---|---|
| α-chitin | 103.9 | 54.6 | 72.9 | 82.8 | 75.4 | 60.5 | 172.6 | 22.5 | Crab shell | Jang, et al. 2004[1] |
| | 104.0 | 54.8 | 73.4 | 82.9 | 75.6 | 60.6 | 173.0 | 22.6 | Crab shell | Kono, et al. 2004[2] |
| | 104.1 | 55.1 | 73.2 | 83 | 75.7 | 60.9 | 173.7 | 22.7 | Crab shell | Kaya, et al. 2017[3] |
| | 104.5 | 55.4 | 73.6 | 83.2 | 76.0 | 61.0 | 173.7 | 23.1 | Crab shell | Tanner, et al. 1990[4] |
| | 104.4 | 55.3 | 73.6 | 83.3 | 76.0 | 61.3 | 174.2 | 23.1 | *I. basta* | Brunner, et al. 2009[5] |
| | 104.6 | 55.6 | 73.7 | 83.6 | 76.0 | 61.1 | 173.0 | 23.1 | Lobster tendon | Tanner, et al. 1990[4] |
| β-chitin | 104.8 | 56.0 | 74.5 | 83.7 | 75.4 | 59.9 | 174.8 | 23.3 | Wet squid pen | Tanner, et al. 1990[4] |
| | 104.2 | 55.2 | 74.8 | 84.1 | 74.8 | 60.9 | 173.1 | 22.5 | Squid | Jang, et al. 2004[1] |
| | 104.1 | 55.2 | 74.2 | 83.4 | 73.6 | 60.8 | 173.6 | 22.8 | Squid pen | Kono, et al. 2004[2] |
| | 105.4 | 55.3 | 73.1 | 84.5 | 75.5 | 59.9 | 175.6 | 22.8 | Diatoms spins | Tanner, et al. 1990[4] |
| | 105.3 | 55.2 | 73.1 | 84.4 | 75.4 | 59.8 | 175.5 | 22.7 | *Tevnia* tube dried | Tanner, et al. 1990[4] |
| | 105.2 | 55.2 | 74.5 | 83.1 | 75.4 | 59.2 | 175.0 | 24.1 | *Tevnia* tube hydrated | Tanner, et al. 1990[4] |
| | 105.3 | 55.8 | 73 | 84.4 | 75.4 | 59.8 | 175.0 | 23.4 | *C. cryptica* | Kolbe, et al. 2021[6] |
| | 105.3 | 55.6 | 73 | 84.4 | 75.4 | 59.8 | 175.8 | 22.7 | *T. rotula* | Brunner, et al. 2009[5] |
| | 104.3 | 55.6 | 75.2 | 84 | 75.2 | 61.7 | 174.4 | 22.9 | Cuttle fish | Kaya, et al. 2017[3] |
| γ-chitin | 103.7 | 54.8 | 73.1 | 82.7 | 75.4 | 61.1 | 173.4 | 22.6 | Lucainade | Jang, et al. 2004[1] |
| | 104.3 | 55.3 | 73.5 | 83 | 75.7 | 61.3 | 173.4 | 22.7 | Cocoon of *O. dubia* | Kaya, et al. 2017[3] |
| Chitosan | 104.7 | 56.8 | 74.1 | 85.7 | 74.1 | 60.7 | / | / | | Heux, et al, 2000[7] |
| | 104.1 | 56.6 | 74.4 | 84.3 | 74.6 | 59.8 | / | / | Crab tendon | Tabeta, et al, 1987[8] |
| | 105.0 | 56.4 | 75.5 | 85.6 | 75.0 | 60.3 | / | / | Crab shell | Tabeta, et al, 1987[8] |

| | | | | | | | | | | |
|---|---|---|---|---|---|---|---|---|---|---|
| | 105.7 | 56.8 | 75 | 84.5 | 75.0 | 60.9 | / | / | Shrimp shell | |
| | 102.7 | 57.3 | 73.9 | 82.7 | 73.9 | 61.9 | / | / | annealed | |
| Type I | 101.0 | 56.0 | 70.8 | 85.1 | 74.8 | 61.7 | / | / | $HNO_3$ | Tabeta, et al, 1987[8] |
| | 99.3 | 55.6 | 70.2 | 84.1 | 74.2 | 62.3 | / | / | $HClO_4$ | |
| | 99.7 | 56.0 | 70.6 | 85.1 | 74.2 | 60.0 | / | / | HBr | |
| | 98.7 | 55.6 | 70.0 | 84.7 | 73.8 | 61.3 | / | / | HI | |
| Type II | 100.5 | 55.2 | 71.2 | 79.4 | 74.4 | 59.5 | / | / | HCl | Tabeta, et al, 1987[8] |
| | 100.3 | 55.2 | 71.0 | 79.1 | 74.4 | 59.8 | / | / | $H_2SO_4$ | |
| | 100.9 | 55.8 | 70.8 | 79.5 | 74.4 | 59.2 | / | / | $H_3PO_4$ | |
| | 99.5 | 55.8 | 70.6 | 78.4 | 74.4 | 58.4 | / | / | $HIO_4$ | |

**Supplementary Table S3**: $^{13}C$ chemical shifts of chitin and chitosan in different fungi observed from 2D $^{13}C$-$^{13}C$ correlation spectra. Alphabetical letters used to denote different allomorphs. The units are in ppm. Ambiguous chitin forms are in italics, ambiguous carbon sites are underlined. Not applicable (/). Unidentified (-).

| Chitin/ Chitosan | C1 | C2 | C3 | C4 | C5 | C6 | CO | $CH_3$ | Experiment methods |
|---|---|---|---|---|---|---|---|---|---|
| *Aspergillus fumigatus* | | | | | | | | | |
| a | 104.3 | 55.7 | 73.2 | 83.9 | 75.2 | 60.7 | 173.9 | 23.3 | |
| b | 104.2 | 55.2 | 73.3 | 82.9 | 75.6 | 60.9 | 174.7 | 22.8 | |
| c | 103.0 | 55.7 | 73.4 | 84.1 | 74.9 | 60.4 | 174.9 | 23.4 | |
| d | 103.6 | 55.1 | 73.4 | 83.3 | 75.8 | 60.4 | 174.4 | 22.7 | $^{13}C$-$^{13}C$ 53 ms CORD |
| e | 104.0 | 54.9 | 73.9 | 82.7 | 76.1 | 60.5 | 173.6 | 22.4 | |
| f | 103.2 | 55.2 | 73.3 | 84.3 | 75.2 | 61.3 | 174.3 | 22.7 | |
| *g* | <u>104.5</u> | 55.1 | <u>74.1</u> | <u>83.7</u> | 75.0 | <u>60.4</u> | <u>174.4</u> | <u>22.4</u> | |
| *h* | <u>103.5</u> | 55.6 | 73.2 | 83.4 | <u>74.9</u> | <u>61.8</u> | <u>175.1</u> | <u>22.8</u> | |
| *Aspergillus nidulans* | | | | | | | | | |
| a′ | 104.2 | 55.4 | 73.6 | 84.1 | 75.6 | 60.2 | 1725 | 22.9 | $^{13}C$-$^{13}C$ 53 ms CORD |
| b′ | 103.2 | 55.7 | 73.9 | 83.6 | 75.9 | 60.5 | 174.6 | 23.1 | |
| c′ | 104.0 | 55.6 | 72.9 | 83.4 | 75.6 | 60.0 | 174.6 | 23.1 | |
| d′ | 102.6 | 54.5 | 73.6 | 83.1 | 75.4 | 60.7 | 173.4 | 22.3 | |
| *Aspergillus sydowii* | | | | | | | | | |
| A | 103.3 | 55.5 | 72.9 | 84.5 | 76.2 | 60.7 | 174.6 | 22.7 | |
| B | 103.5 | 55.2 | 73.4 | 84.4 | 75.9 | 60.0 | 173.4 | 22.7 | |
| C | 103.5 | 55.4 | 73.3 | 83.7 | 75.9 | 60.7 | 173.4 | 22.7 | |
| D | 103.6 | 55.0 | 73.2 | 83.4 | 75.5 | 60.5 | 174.1 | 22.4 | |
| E | 103.2 | 54.8 | 73.5 | 82.5 | 75.2 | 60.9 | 175.1 | 22.4 | $^{13}C$-$^{13}C$ 100 ms DARR |
| chitosan a′ | 102.2 | 55.6 | 74.5 | 80.4 | 74.9 | 60.7 | / | / | |
| chitosan b′ | 101.9 | 55.7 | 72.9 | 80.0 | 74.3 | 60.5 | / | / | |
| chitosan c′ | 102.9 | 55.7 | 72.5 | 79.7 | 75.3 | 60.9 | / | / | |
| chitosan d′ | 101.4 | 55.5 | 73.5 | 79.1 | 75.3 | 61.0 | / | / | |
| *Rhizopus delemar* | | | | | | | | | |
| i | 104.2 | 55.2 | 74.0 | 83.1 | 75.1 | 60.7 | 174.9 | 23.3 | |
| j | 104.3 | 55.2 | 73.8 | 84.2 | 75.0 | 60.8 | 174.9 | 23.6 | $^{13}C$-$^{13}C$ 53 ms CORD |

| | | | | | | | | | |
|---|---|---|---|---|---|---|---|---|---|
| k | 104.2 | 54.9 | 74.0 | 83.1 | 75.1 | 58.9 | 175.7 | 22.8 | |
| chitosan a | 101.9 | 56.0 | 72.5 | 79.6 | 75.0 | 60.3 | / | / | |
| chitosan b | 98.0 | 56.4 | 72.5 | 79.8 | 75.1 | 60.4 | / | / | |
| chitosan c | 98.3 | 57.5 | 70 | 77.0 | 75.4 | 60.4 | / | / | |
| *Candida albicans* | | | | | | | | | |
| l | 102.5 | 55.5 | 73.2 | 84.2 | 75.9 | 60.4 | 175.2 | 22.9 | $^{13}C$-$^{13}C$ 53 ms CORD |
| m | 103.0 | 55.0 | 73.4 | 83.8 | 75.6 | 60.4 | 174.9 | 22.9 | |
| n | 103.9 | 54.6 | 73.1 | 83.0 | 75.7 | 60.3 | 174.2 | 22.5 | |
| o | 103.9 | 54.3 | 73.2 | 82.5 | 75.5 | 60.5 | 173.9 | 22.3 | |
| *Candida auris* | | | | | | | | | |
| l | 102.1 | 55.4 | 73.3 | 84.1 | 75.8 | 60.8 | 175.5 | 22.6 | $^{13}C$-$^{13}C$ 53 ms CORD |
| m | 103.0 | 55.0 | 73.2 | 83.7 | 75.0 | 60.6 | 175.7 | 23.2 | |
| n | 103.5 | 54.4 | 73.2 | 83.1 | 75.6 | 60.7 | 174.3 | 22.6 | |
| o | 103.9 | 54.2 | 73.3 | 82.4 | 75.5 | 60.8 | 174.2 | 22.3 | |

**Supplementary Table S4:** Drug Effect on Chitin types in *A. fumigatus*. Chemical shift difference and RMSD of each type is calculated. The units are in ppm. Δ denotes the difference of with drug and without drug. w/o drug = without drug.

| | | C1 | C2 | C3 | C4 | C5 | C6 | CO | $CH_3$ | RMSD (ppm) |
|---|---|---|---|---|---|---|---|---|---|---|
| Caspofungin | | | | | | | | | | |
| With drug | a | 104.3 | 55.7 | 73.2 | 83.9 | 75.2 | 60.7 | 173.9 | 23.3 | |
| w/o drug | a | 104.2 | 55.2 | 73.2 | 83.7 | 74.8 | 60.6 | 173.1 | 23.5 | |
| | Δ | 0.1 | 0.5 | 0 | 0.2 | 0.4 | 0.1 | 0.9 | -0.2 | 0.4 |
| With drug | b | 104.2 | 55.2 | 73.3 | 82.9 | 75.6 | 60.9 | 174.7 | 22.8 | |
| w/o drug | b | 104.2 | 54.9 | 73.3 | 82.9 | 75.5 | 60.7 | 174.5 | 22.8 | |
| | Δ | 0 | 0.3 | 0 | 0 | 0.1 | 0.2 | 0.2 | 0 | 0.1 |
| With drug | c | 103 | 55.7 | 73.4 | 84.1 | 74.9 | 60.4 | 175.0 | 23.4 | |
| w/o drug | c | 103.1 | 55.4 | 73.1 | 84.1 | 74.1 | 60.3 | 175.0 | 23.1 | |
| | Δ | -0.1 | 0.3 | 0.3 | 0 | 0.8 | 0.1 | 0 | 0.3 | 0.3 |
| With drug | d | 103.6 | 55.1 | 73.4 | 83.3 | 75.8 | 60.4 | 174.4 | 22.7 | |
| w/o drug | d | 103.5 | 55.2 | 73.2 | 83.4 | 75.8 | 60.5 | 173.8 | 22.6 | |
| | Δ | 0.1 | -0.1 | 0.2 | -0.1 | 0 | -0.1 | 0.5 | 0.1 | 0.2 |
| With drug | e | 104.0 | 54.9 | 73.9 | 82.7 | 76.1 | 60.5 | 173.6 | 22.4 | |
| w/o drug | e | 104.0 | 54.9 | 73.7 | 82.6 | 75.5 | 60.6 | 174.9 | 22.3 | |
| | Δ | 0 | 0 | 0.2 | 0.1 | 0.6 | -0.1 | -1.3 | 0.1 | 0.5 |
| With drug | f | 103.2 | 55.2 | 73.3 | 84.3 | 75.2 | 61.3 | 174.3 | 22.7 | |
| w/o drug | f | 103.5 | 55.2 | 73.2 | 84.2 | 75.2 | 60.6 | 174.3 | 22.5 | |
| | Δ | -0.3 | 0 | 0.1 | 0.1 | 0 | 0.7 | 0 | 0.2 | 0.3 |
| Amphotericin B | | | | | | | | | | |
| With drug | a | 104.3 | 55.7 | 73.2 | 83.8 | 75.2 | 60.7 | 173.9 | 23.3 | |
| w/o drug* | a | 103.7 | 55.4 | 73.6 | 83.9 | 74.9 | 60.7 | 173.6 | 22.8 | |
| | Δ | 0.5 | 0.3 | -0.4 | -0.1 | 0.2 | 0 | 0.4 | 0.5 | 0.3 |
| With drug | b | 104.2 | 55.2 | 73.3 | 82.9 | 75.6 | 60.9 | 174.7 | 22.8 | |
| w/o drug | b | 104.0 | 55.2 | 73.4 | 82.9 | 74.9 | 60.6 | 174.1 | 22.7 | |
| | Δ | 0.2 | 0 | -0.1 | 0.0 | 0.7 | 0.3 | 0.6 | 0.1 | 0.3 |

| | | | | | | | | | | |
|---|---|---|---|---|---|---|---|---|---|---|
| With drug | c | 103.0 | 55.7 | 73.4 | 84.1 | 74.9 | 60.4 | 174.9 | 23.4 | |
| w/o drug | c | 103.0 | 56.2 | 73.8 | 83.8 | 74.9 | 60.5 | 175.7 | 23.1 | |
| | Δ | 0 | -0.5 | 0.4 | 0.3 | 0 | -0.1 | -0.7 | 0.3 | 0.3 |
| With drug | d | 103.6 | 55.1 | 73.4 | 83.3 | 75.8 | 60.4 | 174.4 | 22.7 | |
| w/o drug | d | 103.8 | 54.7 | 73.3 | 83.5 | 75.6 | 60 | 174.1 | 22.8 | |
| | Δ | 0.2 | 0.4 | 0.1 | -0.2 | 0.2 | 0.4 | 0.3 | -0.1 | 0.3 |
| With drug | e | 104 | 54.9 | 73.9 | 82.7 | 76.1 | 60.5 | 173.6 | 22.4 | |
| w/o drug | e | 103.4 | 54.7 | 73.6 | 82.8 | 75.9 | 60.3 | 174.4 | 22.5 | |
| | Δ | 0.6 | 0.2 | 0.3 | -0.1 | 0.2 | 0.2 | -0.8 | -0.1 | 0.3 |
| With drug | f | 103.2 | 55.2 | 73.3 | 84.3 | 75.2 | 61.3 | 174.3 | 22.7 | |
| w/o drug | f | 103.3 | 55.6 | 73.4 | 84.2 | 75 | 60.8 | 174.3 | 23.0 | |
| | Δ | -0.1 | -0.4 | -0.1 | 0.1 | 0.2 | 0.5 | 0 | -0.4 | 0.3 |

**Supplementary Table S5:** Salt effect on chitin types in *A. sydowii*. Δ denotes the chemical shift difference of samples prepared using 0.5 M and 2.0 M NaCl. RMSD indicates the chemical shift difference between each chitin type in 0.5 M and 2 M NaCl *A. sydowii* samples (ppm).

| | | C1 | C2 | C3 | C4 | C5 | C6 | CO | $CH_3$ | RMSD (ppm) |
|---|---|---|---|---|---|---|---|---|---|---|
| 0.5 M | A | 103.3 | 55.5 | 72.9 | 84.5 | 76.2 | 60.7 | 174.6 | 22.7 | |
| 2 M | A | 103.4 | 55.2 | 73.0 | 84.4 | 76.0 | 61.3 | 175.0 | 23.1 | |
| | Δ | -0.1 | 0.3 | -0.1 | 0.1 | 0.2 | -0.6 | -0.4 | -0.4 | 0.3 |
| 0.5 M | B | 103.5 | 55.2 | 73.4 | 84.4 | 75.9 | 60.0 | 173.4 | 22.7 | |
| 2 M | B | 103.3 | 55.1 | 73.2 | 83.8 | 75.8 | 60.2 | 174.7 | 22.3 | |
| | Δ | 0.2 | 0.2 | 0.2 | 0.6 | 0.1 | -0.2 | -1.3 | 0.4 | 0.6 |
| 0.5 M | C | 103.5 | 55.4 | 73.3 | 83.7 | 76.0 | 60.7 | 173.4 | 22.7 | |
| 2 M | C | 103.5 | 55.1 | 73.3 | 83.3 | 75.6 | 60.8 | 173.4 | 22.3 | |
| | Δ | 0 | 0.3 | 0 | 0.4 | 0.4 | -0.1 | 0 | 0.5 | 0.3 |
| 0.5 M | D | 103.6 | 55 | 73.2 | 83.4 | 75.5 | 60.5 | 174.1 | 22.4 | |
| 2 M | D | 103.6 | 55 | 73.2 | 83.0 | 75.3 | 60.8 | 174.2 | 22.5 | |
| | Δ | 0 | 0 | 0 | 0.3 | 0.2 | -0.3 | -0.1 | -0.1 | 0.2 |
| 0.5 M | E | 103.2 | 54.8 | 73.5 | 82.5 | 75.2 | 60.9 | 175.1 | 22.4 | |
| 2 M | E | 103.6 | 54.4 | 73.3 | 82.1 | 75.3 | 60.9 | 174.4 | 22.4 | |
| | Δ | -0.4 | 0.4 | 0.2 | 0.4 | -0.1 | 0 | 0.7 | 0.0 | 0.4 |

**Supplementary References**


1. Jang, M. K.; Kong, B. G.; Jeong, Y. I.; Lee, C. H.; Nah, J. W., Physicochemical characterization of α-chitin, β-chitin, and γ-chitin separated from natural resources. *Journal of Polymer Science Part A: Polymer Chemistry* **2004,** *42* (14), 3423-3432.
2. Kono, H., Two-dimensional magic angle spinning NMR investigation of naturally occurring chitins: Precise 1H and 13C resonance assignment of α-and β-chitin. *Biopolymers: Original Research on Biomolecules* **2004,** *75* (3), 255-263.
3. Kaya, M.; Mujtaba, M.; Ehrlich, H.; Salaberria, A. M.; Baran, T.; Amemiya, C. T.; Galli, R.; Akyuz, L.; Sargin, I.; Labidi, J., On chemistry of γ-chitin. *Carbohydrate polymers* **2017,** *176*, 177-186.
4. Tanner, S. F.; Chanzy, H.; Vincendon, M.; Roux, J. C.; Gaill, F., High-Resolution Solid-State C-13 Nuclear-Magnetic-Resonance Study of Chitin. *Macromolecules* **1990,** *23* (15), 3576-3583.
5. Brunner, E.; Ehrlich, H.; Schupp, P.; Hedrich, R.; Hunoldt, S.; Kammer, M.; Machill, S.; Paasch, S.; Bazhenov, V.; Kurek, D., Chitin-based scaffolds are an integral part of the skeleton of the marine demosponge Ianthella basta. *Journal of structural biology* **2009,** *168* (3), 539-547.
6. Kolbe, F.; Ehren, H. L.; Kohrs, S.; Butscher, D.; Reiß, L.; Baldus, M.; Brunner, E., Solid-state NMR spectroscopic studies of 13 C, 15 N, 29 Si-enriched biosilica from the marine diatom Cyclotella cryptica. *Discover Materials* **2021,** *1* (1), 1-12.
7. Heux, L.; Brugnerotto, J.; Desbrieres, J.; Versali, M. F.; Rinaudo, M., Solid state NMR for determination of degree of acetylation of chitin and chitosan. *Biomacromolecules* **2000,** *1* (4), 746-751.
8. Saito, H.; Tabeta, R.; Ogawa, K., High-resolution solid-state $^{13}C$ NMR study of chitosan and its salts with acids: conformational characterization of polymorphs and helical structures as viewed from the conformation-dependent $^{13}C$ chemical shifts. *Macromolecules* **1987,** *20* (10), 2424-2430.
